# Onset and progression of postmortem histological changes in the central nervous system of RccHan^™^: WIST rats

**DOI:** 10.3389/fvets.2024.1378609

**Published:** 2024-05-21

**Authors:** Klaus Weber, Anna Domènech, Kristel Kegler, Robert Kreutzer, Francisco José Mayoral, Yoshimasa Okazaki, Paula Ortega, Laura Polledo, Tanja Razinger, Olivia Kristina Richard, Raúl Sanchez, Nils Warfving, Raquel Vallejo, Ricardo de Miguel

**Affiliations:** ^1^AnaPath Services GmbH, Liestal, Switzerland; ^2^AnaPath Research S.A.U., Barcelona, Spain

**Keywords:** autolysis, postmortem, rat, artifact, delayed fixation, decomposition, brain, central nervous system

## Abstract

Death initiates a cascade of physiological and biochemical alterations in organs and tissues, resulting in microscopic changes that challenge the histopathological evaluation. Moreover, the brain is particularly susceptible to artifacts owing to its unique composition and its location within the cranial vault. The aim of this study was to compile and illustrate the microscopic changes in the central nervous system (CNS) of rats subjected to delayed postmortem fixation. It also scrutinizes the influence of exsanguination and cooling methods on the initiation and progression of these alterations. Twenty-four Wistar Han outbred rats (RccHan^™^: WIST) were sacrificed and stored either at room temperature (18–22°C) or under refrigeration (2–4°C). Necropsies were conducted at different time points postmortem (i.e., 0.5 h, 1 h, 4 h, 8 h, 12 h, 24 h, 36 h, 48 h, 7 days and 14 days). Brain sections underwent simultaneous digital evaluation by 14 pathologists until a consensus was reached on terminology, key findings, and intensity levels. Microscopic observations varied among cell types. Glial cells were similarly affected throughout the CNS and showed pericellular halo, chromatin condensation and nuclear shrinkage. Neurons showed two types of postmortem changes as most of them showed progressive shrinkage, cytoplasmic dissolution and karyorrhexis whereas others acquired a dark-neuron-like appearance. Neuronal changes showed marked differences among neuroanatomical locations. Additional postmortem changes encompassed: granulation and microcavitation in neuropil and white matter; retraction spaces; detachment of ependyma, choroid plexus, and leptomeninges. Severity of findings after 48 h at room temperature was higher than after seven days under refrigeration and similar to or slightly lower than after 14 days under refrigeration. No clear differences were observed related to the sex or weight of the animals or their exsanguination status. This work elucidates the onset and progression of autolytic changes in the brains of Wistar Han rats, offering insights to accurately identify and enhance the histopathological evaluation.

## 1 Introduction

Somatic or systemic animal death is defined as the cessation of the vital functions of the brain, heart, and lungs. The halt of blood flow in these organs triggers a sequence of physical and chemical alterations in tissues and cells leading to postmortem changes (1), which can be categorized as discrete phenomena including algor mortis, livor mortis, rigor mortis, desiccation, decomposition, and mummification (2, 3). These phenomena can manifest concurrently and are influenced by intrinsic factors and environmental conditions, such as temperature, humidity, oxygen tension and invertebrate activity (3, 4).

Body decomposition is the result of two distinct processes: autolysis and putrefaction. Autolysis is the self-digestion of cells by the action of their own enzymes. Release and activation of these enzymes are consequences of disrupted cellular homeostasis and loss of cellular membrane integrity. It is mainly caused by the exhaustion of ATP and pH decrease related to the lack of oxygen, the failure of oxidative phosphorylation and the counterbalance activation of anerobic glycolysis. In some organs, autolysis creates the ideal conditions for bacterial proliferation leading to putrefaction (3).

Histopathological evaluation of the central nervous system (CNS) in animals and humans is challenging due to its anatomical complexity and the wide range of pathological entities (5). Moreover, the structural components of the brain and its location within the calvarium make this organ prone to artifacts that can be misinterpreted as real findings by forensic, diagnostic and toxicologic pathologists. Autolysis and putrefaction are widely known artifacts of the CNS in unproperly fixed specimens. However, there is scarce information to properly identify them during routine histological evaluation. This highlights the need for a comprehensive description and illustration of these changes to avoid the distortion or misidentification of real findings (3). The aim of this study was to systematically compile and illustrate the microscopic changes in rat brains subsequent to delayed postmortem fixation. Furthermore, we aimed to analyze the potential effect of exsanguination, cooling, air ventilation considering the variables of sex and body weight in the onset and progression of these findings.

## 2 Materials and methods

### 2.1 Study design

All animals employed in the present study were surplus naïve animals from regulatory studies approved by the Ethical Committee of the Generalitat de Catalunya (Departament d’Acció Climática, Alimentació y Agenda Rural) and licensed under ref. 10,832. Requirements of the Spanish Policy for Animal Protection (RED118/2021 and RED1386/2018) and the European Union Directive 2010/63 on the protection of experimental animals were always fulfilled. In total, 24 Wistar Han outbred rats (RccHan^®^:WIST) were sacrificed by intraperitoneal overdose of phenobarbital sodium (Dolethal^®^, Vetoquinol).

Sixteen carcasses were stored uncovered under controlled room temperature (RT; 18–22°C) to mimic the conditions of animal housing rooms. Of these, eight carcasses were exsanguinated whereas the other eight animals were not exsanguinated. Mimicking standard protocols in regulatory studies, exsanguination was performed immediately after death by bilateral section of brachial plexus and abdominal aorta after ventral cavity exposure, extravasated blood was aspirated using a suction device until bleeding stopped. Rats were necropsied at different time points after euthanasia (i.e., 0.5, 1, 4, 8, 12, 24, 36 and 48 h). At each time point, an exsanguinated and a non-exsanguinated animal were necropsied.

Eight additional non-exsanguinated carcasses were stored in a refrigerator (2–4°C). Of these, four carcasses were placed in a perforated cardboard box and the other four carcasses in an opaque plastic bag, thereby mimicking long-term storage conditions with and without air circulation, respectively. These animals were necropsied seven and 14 days after euthanasia. At each time point, two animals (one male and one female) stored in the cardboard box and two (one male and one female) from within the plastic bag were necropsied. The distribution of sexes for the different study conditions are provided in Supplementary Table 1.

### 2.2 Histoprocessing and histopathology evaluation

**Figure 1:**
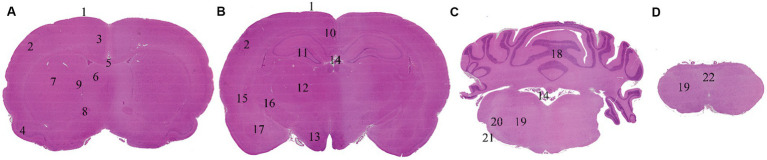
Brain levels and neuroanatomical locations evaluated. Non-exsanguinated animal. 0.5 h time point. Trimming and levels named following Bolon et al. (6) recommended practices. **(A)** Level 2. **(B)** Level 3. **(C)** Level 6. **(D)** Level 7. 1: Leptomeninges. 2: Frontoparietal cortex. 3: Cingulate cortex. 4: Piriform cortex. 5: Corpus callosum. 6: Septal nuclei. 7: Caudate-putamen. 8: Anterior commissure. 9: Ependyma. 10: Retrosplenial cortex. 11: Hippocampus. 12: Thalamus. 13: Hypothalamus. 14: Choroid plexus. 15: Capsula externa. 16: Capsula interna. 17: Amygdaloid nuclei. 18: Cerebellum. 19: Reticular nuclear area. 20: Lateral trigeminal tract. 21: Trapezoid body. 22: Predorsal bundle.

Sampling, trimming and histology processing of the brain was performed following the Society of Toxicologic Pathology best practices recommendations (6). Briefly, brain sampling started with a midline incision of the scalp followed by dissection of the muscles to reveal the calvarium. Subsequently, bone removal was executed in a caudal to cranial direction utilizing rongeurs. Brain was extracted employing surgical forceps and fixed by immersion in neutral phosphate-buffered 10% formalin for 48 to 72 h, maintaining a formalin to organ ratio of 1:20. At trimming, coronal sections were performed, embedded in paraffin, cut at a nominal thickness of 2–4 μm and stained with hematoxylin–eosin. Levels 2, 3, 6 and 7 of Bolon et al. (6) were selected for evaluation, including the following neuroanatomical locations: frontoparietal cortex, retrosplenial cortex, cingulate cortex, piriform cortex, corpus callosum, caudate-putamen (globus pallidus and bed nucleus of the stria terminalis), septal nuclei, anterior commissure, hippocampus, thalamus, hypothalamus, amygdaloid nuclei, capsula interna, capsula externa, cerebellum and pons (predorsal bundle, trapezoid body, lateral trigeminal tract, reticular nuclear area), ependyma, choroid plexus and leptomeninges (Figure 1).

Samples were scanned with a 20x objective (200x magnification) by an Olympus Slideview VS200 slide scanner coupled to a *VS*-264C camera. Digital slides were analyzed concurrently by 14 pathologists until a shared consensus was reached on the most relevant postmortem histological changes, their intensity degree, and the terminology employed. The team of pathologists was composed of professionals with different levels of expertise and educational backgrounds including ECVP, JCVP or JSTP board-certified pathologists.

Descriptive terminology, avoiding the use of specific diagnostic terms, was systematically applied to characterize the main histological changes observed. Postmortem changes were scored from 0 to 3 (i.e., 0 = absent to minimal; 1 = mild; 2 = moderate; 3 = marked) based on their distribution, extension, and intensity degree.

## 3 Results

### 3.1 Terminology

The terms employed to describe the postmortem alterations observed in the brain are displayed in Table 1. A concise explanation of each term and alternative terms employed in the literature are included. For practical purposes, some of these findings were evaluated together in same cell types of different neuroanatomical locations or merged after evaluation to be reported in summary tables.

**Table 1:** Description of postmortem histological findings in the brain of Wistar Han rats and the cell type or neuroanatomical region affected.

Finding	Location	Description
Pericellular halo	Glial cells: multiple locations^a^ Neurons: granule cells of the cerebellum	Optically empty space surrounding the nucleus of the cell. Associated with either contraction of the cytoplasm and/or retraction of the surrounding structures (i.e., neuropil or white matter).
Nuclear shrinkage	Glial cells: multiple locations^a^ Leptomeninges	Decreased nuclear size. Associated with chromatin condensation at earlier time points and progressive loss of chromatin at later time points.
Chromatin condensation	Glial cells: multiple locations^a^ Neurons: granule cells of the cerebellum Leptomeninges	Chromatin clumping and agglomeration leading to highly compacted and intensely stained nuclei.
Nuclear fading	Neurons: multiple locations^b^ Ependyma Choroid plexus	Decreased nuclear basophilia and less discernible nucleus. Described in other publications as “Loss of nuclear basophilic staining” (7).
Cytoplasmic dissolution	Neurons: multiple locations^b^	Increased granulation and/or flocculent or ground-glass appearance of the cytoplasm with loss of cell borders. Associated with decreased staining affinity and progressive cytoplasmic shrinkage and loss.
Dark neurons, amount	Frontoparietal cortex Cingulate cortex Retrosplenial cortex Piriform cortex Hippocampus Hypothalamus Purkinje cells	Relative amount of hypercontracted and deeply stained (amphophilic/basophilic) neurons.
Dark neurons, staining intensity	Cortex Hippocampus Hypothalamus	Staining intensity (amphophilic/basophilic) of the perikaryon of hypercontracted neurons.
Granulation	Neuropil: multiple locations^c^ White matter: cerebellum	Aggregation of substances leading to a uniformly ground glass appearance of the extracellular matrix.
Microcavitation	Neuropil: multiple locations^c^ White matter: cerebellum, predorsal bundle, lateral trigeminal tract	Multifocal to coalescing extracellular areas of optically empty irregular spaces, leading to progressive fragmentation, rupture, and loss. In the molecular layer of the encephalon and cerebellum this was associated with fragmentation and loss of the external surface.
Retraction spaces	Blood vessels: cortex, hippocampus Cerebellum: Purkinje cell layer Hippocampus: blades of dentate gyrus	Extracellular optically empty spaces with vacuole-like appearance inducing the separation of neuronal layers or blood vessels from surrounding parenchyma. Previously described as a fixation artifact (8).
Prominent axons	Cingulate cortex; Hippocampus: CA1/CA2 regions	Longitudinal, faintly stained axons that stand out over the surrounding neuropil.
Detachment	Ependyma (from neuropil) Choroid plexus (from capillaries) Leptomeninges	Separation of epithelial lining cells from the underlying structures.
Cilial clumping/loss	Ependyma Choroid plexus	Disorganization of cilia with fusion and/or loss in the ventricular/ependymal lumen.
Cytoplasmic leaking	Choroid plexus	Amorphous pale extracellular eosinophilic material in proximity to choroid plexus epithelial cells
Axonal splitting/dissolution	White matter: predorsal bundle, lateral trigeminal tract	Fragmentation of axons of prominent nerve fibers and progressive loss.
Myelin sheath dilation	White matter: predorsal bundle, lateral trigeminal tract	Increased diameter of myelin sheath in prominent nerve fibers.

a Glial cells in the following neuroanatomical compartments: cortex, corpus callosum, caudate-putamen, septal nuclei, anterior commissure, thalamus, hypothalamus, capsula interna, capsula externa, cerebellum, reticular nuclear area.

b Neurons in the following neuroanatomical compartments: cortex, caudate-putamen, septal nuclei, thalamus, hypothalamus, amygdaloid nucleus, capsula interna, capsula externa, Purkinje cells, reticular nuclear area.

c Neuropil in the following neuroanatomical compartments: cortex, caudate-putamen, anterior commissure, hypothalamus, capsula interna, capsula externa.

### 3.2 Onset and progression of postmortem histological changes

Postmortem histological changes in the brain showed morphological differences between neurons and glial cells and different onset and progression among neuroanatomical compartments. Additionally, regional differences were found in the brain, mainly at latter time points where marked microcavitation/fragmentation of the cerebral cortex and the cerebellar folia were recorded. A summary of the most relevant changes in non-exsanguinated animals stored at RT is displayed in Table 2. The complete histological evaluation of these animals is displayed in Supplementary Table S2. Of note, all animals were healthy and no pathological findings suggestive of disease were observed either in the brain or in other evaluated organs of these animals.

**Table 2:** Summarized postmortem histological changes in the brain of non-exsanguinated outbred RccHan^™^: WIST rats stored at room temperature (18–22°C) and necropsied at different time points after death.

	Time after death							
	0.5 h	1 h	4 h	8 h	12 h	24 h	36 h	48 h
**Glial cells**^**a**^								
Cortex	1.0	1.0	1.0	1.3	2.0	2.7	3.0	3.0
Corpus callosum	1.0	1.0	1.0	2.0	2.0	2.7	2.7	3.0
Caudate-putamen	–	1.0	1.0	1.3	1.7	2.0	2.3	3.0
Septal nuclei	–	1.0	1.0	1.0	1.7	2.7	3.0	3.0
Anterior commissure	1.0	1.0	1.5	1.7	1.3	1.7	2.3	2.7
Hippocampus	–	1.0	1.0	1.3	1.3	2.3	2.3	2.7
Thalamus	–	1.0	1.0	1.3	1.3	2.3	2.3	2.7
Hypothalamus	–	1.0	1.0	1.3	1.7	2.3	2.3	2.7
Capsula interna & Capsula externa	–	–	1.0	1.3	1.3	2.3	2.7	2.7
Cerebellum	–	–	1.0	1.3	1.7	2.0	2.7	2.7
Reticular nuclear area	–	–	1.0	1.3	1.3	2.3	2.3	2.7
*Average*	0.3	0.7	1.0	1.4	1.6	2.3	2.5	2.8
**Neurons**^**b**^								
Cortex	–	–	–	–	1.5	1.5	2.5	3.0
Caudate-putamen	–	–	–	–	1.0	2.0	3.0	3.0
Septal nuclei	–	1.0	1.0	1.0	1.5	1.5	2.5	3.0
Hippocampus	–	–	1.0	1.0	1.0	1.5	1.5	1.5
Thalamus	–	–	–	1.0	1.0	1.5	1.5	2.5
Hypothalamus	–	–	1.0	1.0	2.0	2.5	2.5	2.5
Amygdaloid nuclei	–	–	–	1.0	1.5	2.5	2.5	2.5
Capsula interna & Capsula externa	–	–	2.0	1.5	2.0	2.5	2.5	3.0
Cerebellum, Purkinje cells	–	–	–	1.0	1.0	2.0	2.0	2.0
Reticular nuclear area	–	–	–	1.5	1.5	2.0	2.5	3.0
*Average*	0.0	0.1	0.5	0.9	1.4	2.0	2.3	2.6
**Dark neurons**								
Frontoparietal cortex, amount	3.0	2.0	3.0	3.0	3.0	3.0	3.0	3.0
Cingulate cortex, amount	–	–	–	–	–	1.0	2.0	2.0
Retrosplenial cortex, amount	–	1.0	2.0	1.0	2.0	2.0	2.0	2.0
Piriform cortex, amount	2.0	2.0	2.0	2.0	2.0	3.0	3.0	3.0
Hippocampus, amount	2.0	2.0	2.0	2.0	2.0	2.0	2.0	2.0
Thalamus, amount	1.0	1.0	2.0	1.0	1.0	2.0	1.0	1.0
Hypothalamus, amount	3.0	2.0	2.0	2.0	2.0	2.0	2.0	2.0
Purkinje cells, amount	–	–	–	1.0	1.0	2.0	2.0	2.0
Staining intensity, mean^c^	3.0	3.0	2.5	2.0	2.0	2.0	1.75	1.5
*Average*	1.6	1.4	1.7	1.6	1.7	2.1	2.1	2.1
**Neuropil**^**d**^								
Cortex	–	1.0	1.0	1.0	2.0	2.0	3.0^F^	3.0^F^
Caudate-putamen	–	–	–	1.0	1.0	2.0	2.0	2.0
Anterior commissure	–	–	–	1.0	1.0	1.0	1.0	2.0
Hypothalamus	–	–	–	1.0	1.0	2.0	2.0	2.0
Capsula interna & Capsula externa	–	–	–	1.0	1.0	2.0	2.0	3.0
*Average*	0.0	0.2	0.2	1.0	1.2	1.8	2.0	2.4
**White matter**^**e**^								
Cerebellum	–	–	–	1.0	1.0	1.0	2.0	3.0
Trapezoid body	1.0	1.0	2.0	2.0	3.0	3.0	3.0	3.0
Predorsal bundle	–	–	–	–	1.0	1.0	2.0	2.5
Lateral trigeminal tract	–	–	–	1.0	2.0	2.5	3.0	3.0
*Average*	0.3	0.3	0.5	1.0	1.8	1.9	2.5	2.9
**Other features**								
Retraction spaces, vessels, cortex	1.0	1.0	1.0	2.0	2.0	2.0	3.0	2.0
Retraction spaces, vessels, hippocampus	1.0	1.0	1.0	2.0	2.0	2.0	2.0	2.0
Retraction spaces, blades of dentate gyrus	1.0	1.0	1.0	1.0	2.0	1.0	2.0	3.0
Retraction spaces, Purkinje cells	1.0	1.0	1.0	2.0	2.0	2.0	3.0	3.0
Prominent axons, cingulate cortex	3.0	3.0	2.0	2.0	1.0	1.0	1.0	1.0
Prominent axons, CA1/CA2 regions	3.0	3.0	3.0	2.0	2.0	2.0	1.0	1.0
**Ependyma**								
Nuclear fading	–	–	–	1.0	1.0	1.0	2.0	2.0
Detachment, from neuropil	1.0	1.0	2.0	2.0	2.0	2.0	2.0^R^	2.0^R^
Cilial clumping/loss	–	–	–	1.0	1.0	2.0	3.0	3.0
*Average*	0.3	0.3	0.7	1.3	1.3	1.7	2.3	2.3
**Choroid plexus**								
Nuclear fading	–	–	–	1.0	1.0	1.0	2.0	2.0
Detachment, from capillaries	–	–	–	1.0	1.0	2.0	3.0	2.0
Cilial clumping/loss & cytoplasmic leaking	–	–	1.0	1.0	1.0	2.0	3.0	3.0
*Average*	0.0	0.0	0.3	1.0	1.0	1.7	2.7	2.3
**Leptomeninges**								
Chromatin condensation	–	–	–	–	–	1.0	1.0	1.0
Detachment	1.0	1.0	1.0	1.0	1.0	1.0	1.0	2.0
*Average*	0.5	0.5	0.5	0.5	0.5	1.0	1.0	1.5

a Mean severity of the findings in glial cells: pericellular halo; nuclear shrinkage; chromatin condensation.

b Mean severity of the findings in neurons: cytoplasmic dissolution; nuclear fading.

c Mean severity of the staining intensity in: brain cortex; hippocampus; thalamus; hypothalamus.

d Mean severity of the findings in neuropil: granulation; microcavitation.

e Mean severity of the findings in white matter: granulation; microcavitation; myelin sheath dilation.

^F^ Fragmentation of the molecular layer of the cerebral cortex.^R^ Rupture and discontinuity.

**Figure 2:**
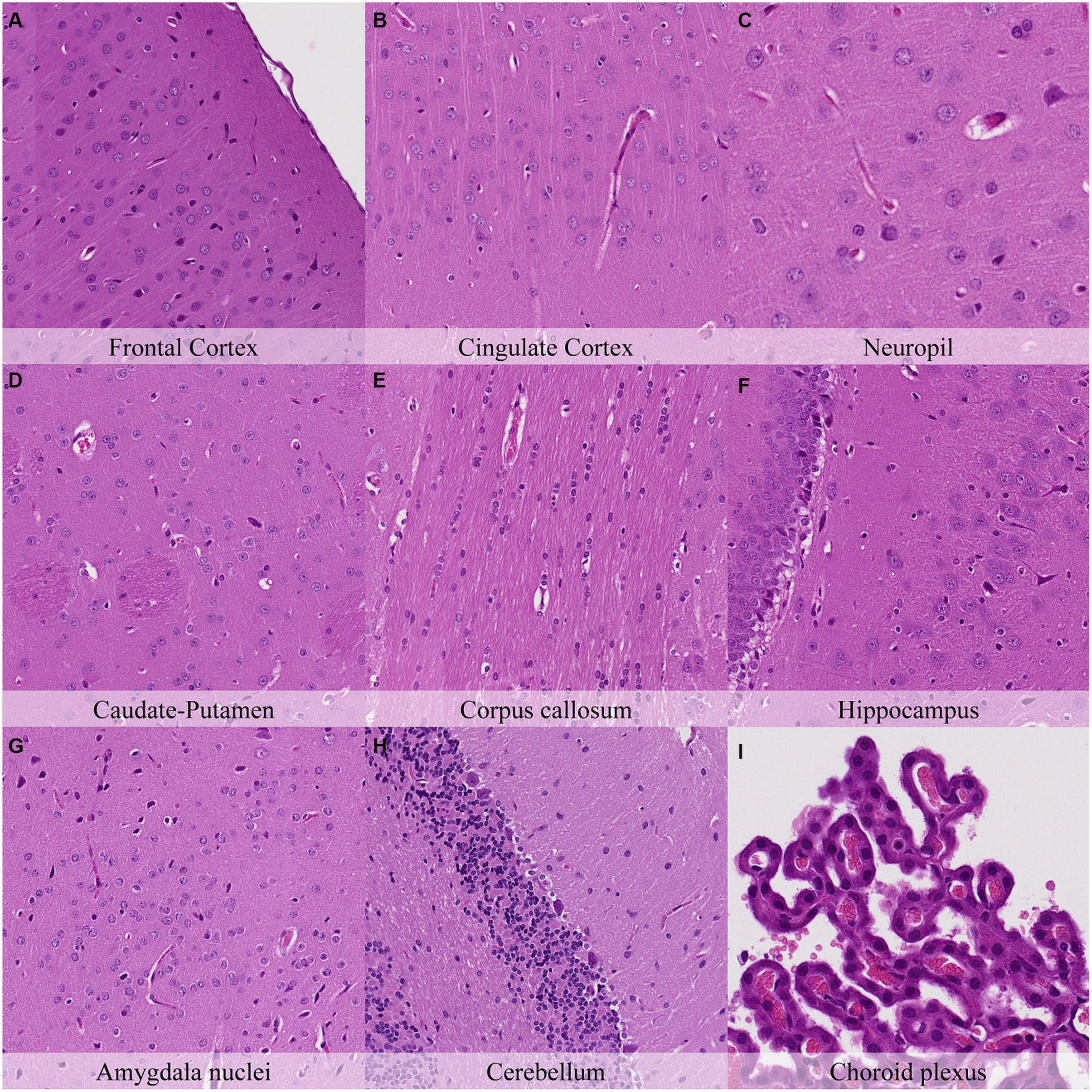
Postmortem microscopic changes in the brain after a delayed postmortem fixation of 0.5 h. Carcass stored at room temperature (18–22°C). Hematoxylin–eosin stain. **(A)** Frontal cortex, 100x magnification. **(B)** Cingulate cortex, 100x magnification. **(C)** Neuropil, 200x magnification. **(D)** Caudate-putamen, 100x magnification. **(E)** Corpus callosum, 100x magnification. **(F)** Hippocampus, 100x magnification. **(G)** Amygdala nuclei, 100x magnification. **(H)** Cerebellum, 100x magnification. **(I)** Choroid plexus, 200x magnification.

Microscopical changes present at 30 min postmortem included: retraction spaces around blood vessels; prominent axons in the cingulate cortex and in CA1/CA2 subfields of the hippocampus; and detachment of ependyma/leptomeninges with exposure of subjacent neuropil (Figure 2; Supplementary Figure S2). Moreover, dark neurons were abundant in some regions (i.e., frontoparietal and piriform cortex, hippocampus, hypothalamus, and Purkinje cell layer) whereas they were scarce to absent in others (i.e., cingulate and retrosplenial cortex and thalamus). Glial cells showed a minimal clear pericellular halo in the cortex, corpus callosum and anterior commissure.

**Figure 3:**
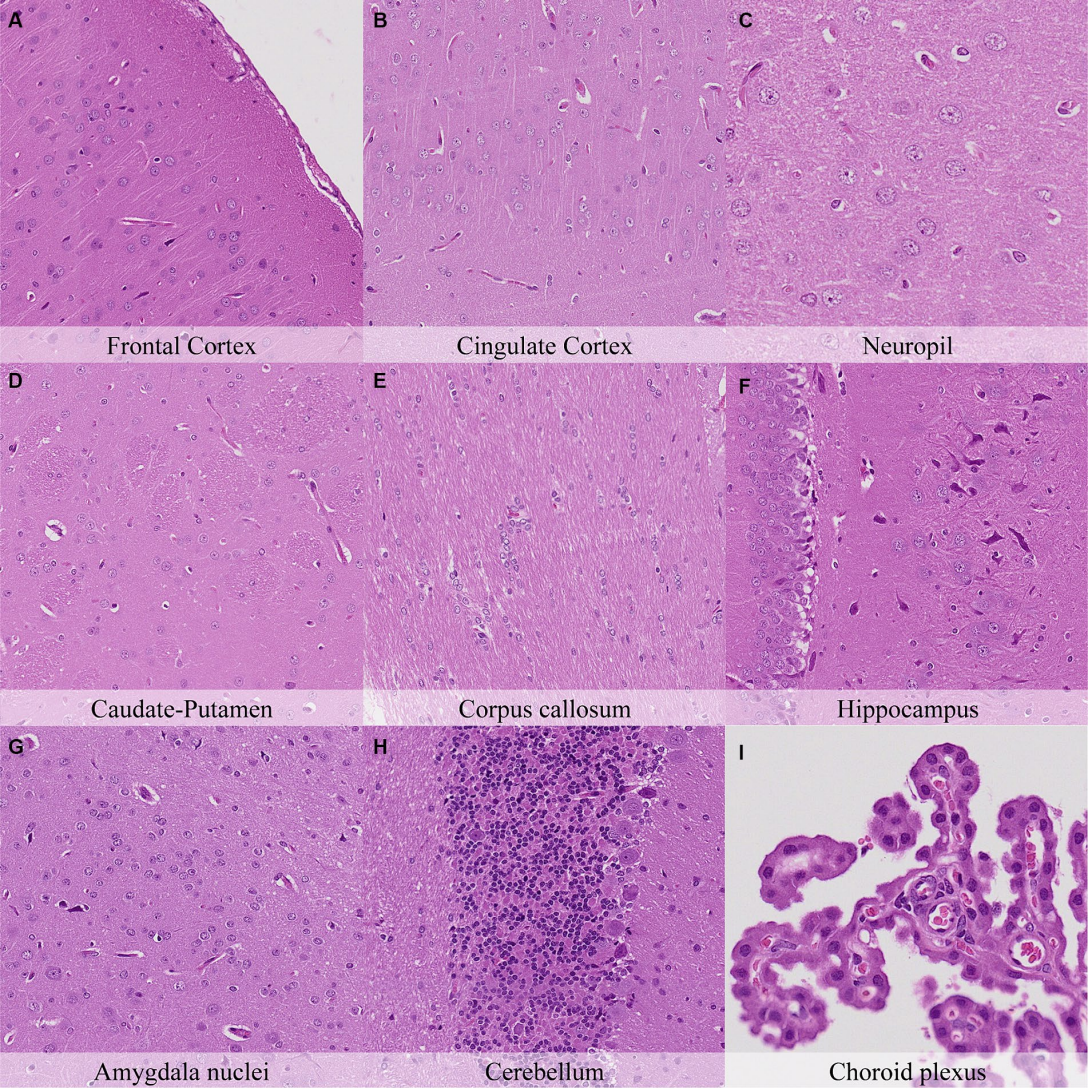
Postmortem microscopic changes in the brain after a delayed postmortem fixation of 1 h. Carcass stored at room temperature (18–22°C). Hematoxylin–eosin stain. **(A)** Frontal cortex, 100x magnification. **(B)** Cingulate cortex, 100x magnification. **(C)** Neuropil, 200x magnification. **(D)** Caudate-putamen, 100x magnification. **(E)** Corpus callosum, 100x magnification. **(F)** Hippocampus, 100x magnification. **(G)** Amygdala nuclei, 100x magnification. **(H)** Cerebellum, 100x magnification. **(I)** Choroid plexus, 200x magnification.

One hour postmortem, additional changes were observed including: pericellular halo around glial cells in the caudate-putamen, septal nuclei, hippocampus, thalamus and hypothalamus (Figure 3; Supplementary Figure S3); Mild granulation of the neuropil and microcavitation in the outermost neuronal layer (i.e., molecular layer) on the cerebral cortex; and minimal cytoplasmic dissolution in the neurons of the septal nuclei.

**Figure 4:**
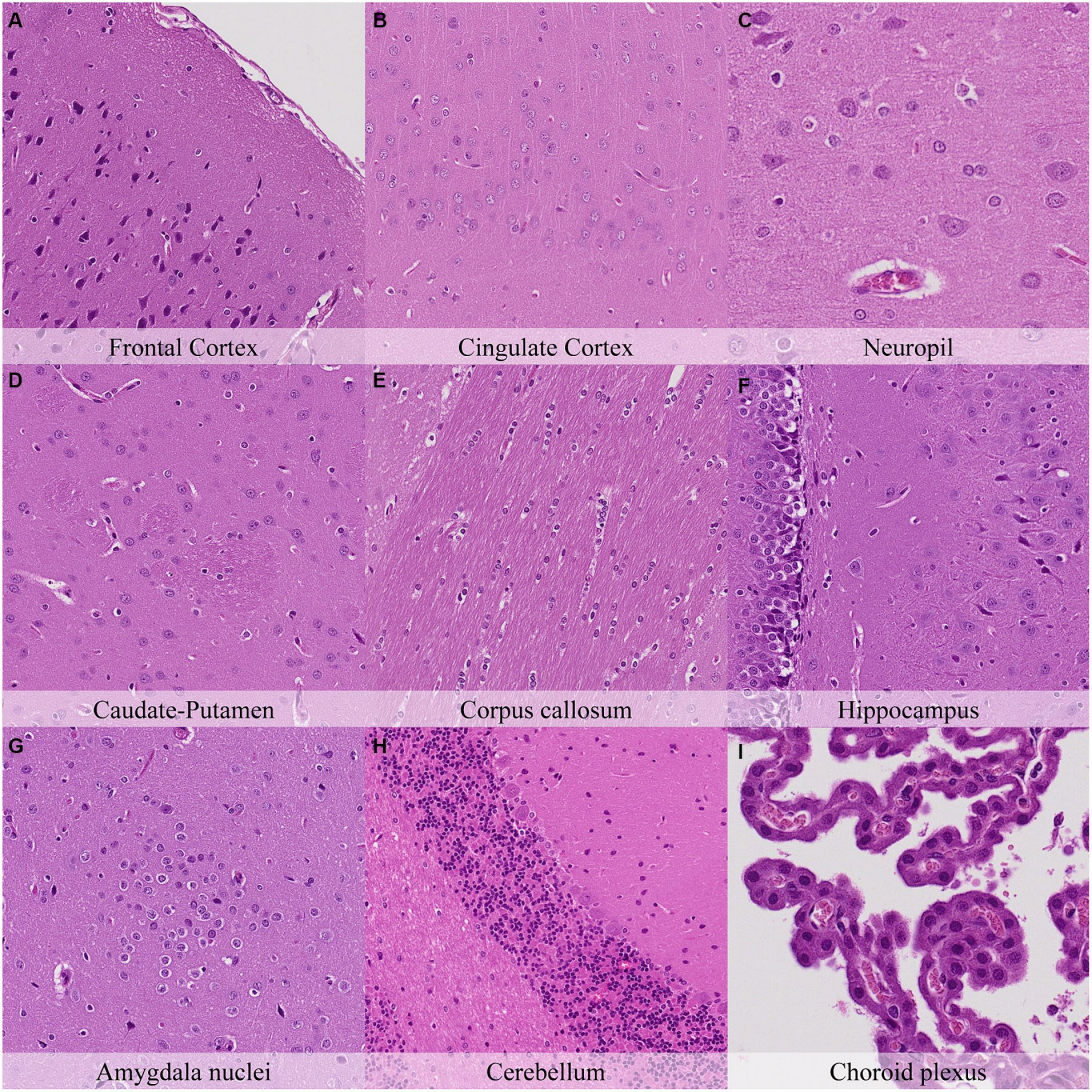
Postmortem microscopic changes in the brain after a delayed postmortem fixation of 4 h. Carcass stored at room temperature (18–22°C). Hematoxylin–eosin stain. **(A)** Frontal cortex, 100x magnification. **(B)** Cingulate cortex, 100x magnification. **(C)** Neuropil, 200x magnification. **(D)** Caudate-putamen, 100x magnification. **(E)** Corpus callosum, 100x magnification. **(F)** Hippocampus, 100x magnification. **(G)** Amygdala nuclei, 100x magnification. **(H)** Cerebellum, 100x magnification. **(I)** Choroid plexus, 200x magnification.

Four hours postmortem, glial cells displayed nuclear shrinkage and/or chromatin condensation in all neuroanatomical locations (Figure 4; Supplementary Figure S4). Axons in the cingulate cortex were slightly less prominent than at previous time points. Neurons of the capsula interna/externa showed moderate cytoplasm dissolution, and neurons in the granule cell layer of the cerebellar folia showed a mild increase in chromatin condensation. Mild cilial clumping and cytoplasmic leakage were recorded in the choroid plexus. Dark neurons showed a similar distribution but with a minimal decrease in staining intensity in contrast to the initial time point (Supplementary Figure S4).

**Figure 5:**
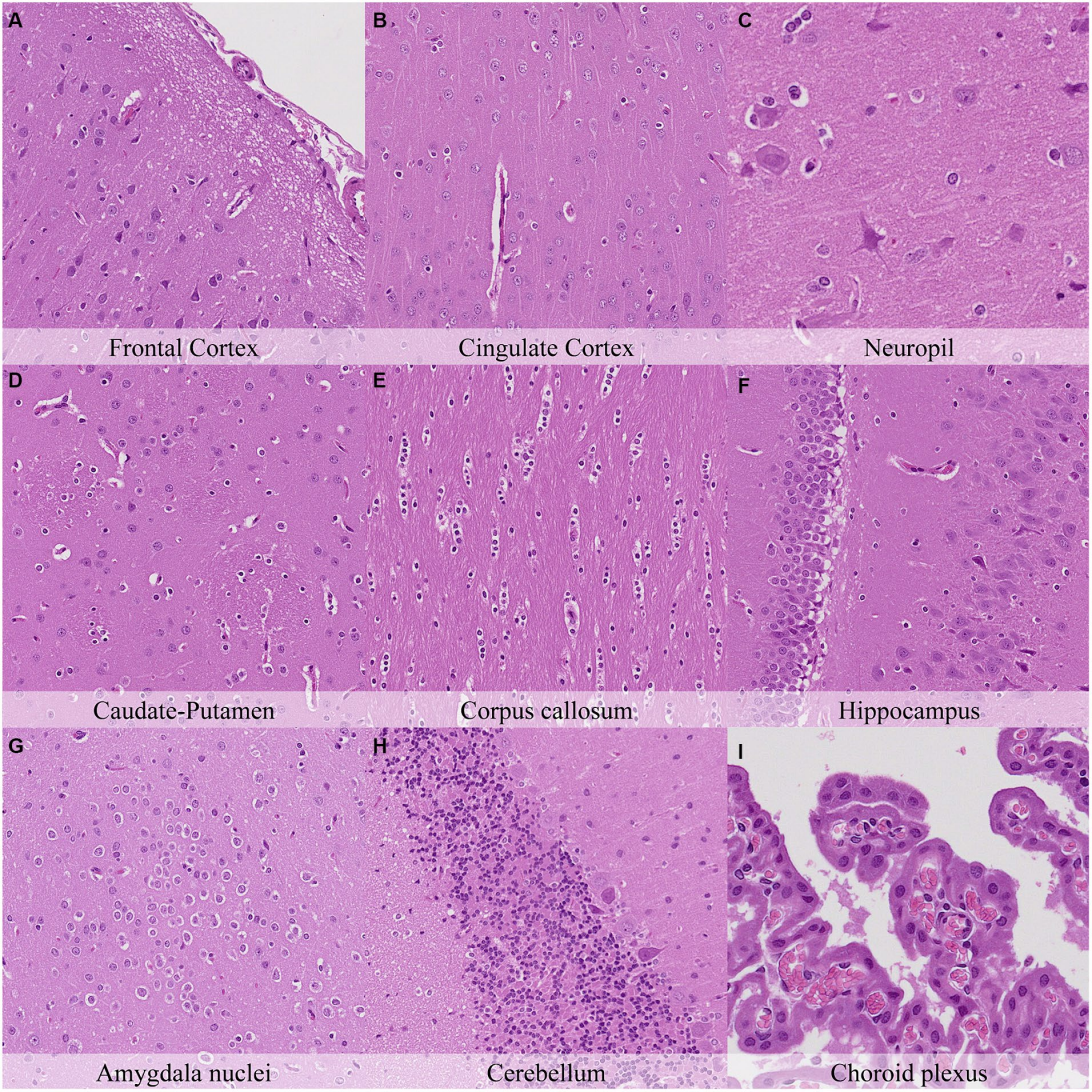
Postmortem microscopic changes in the brain after a delayed postmortem fixation of 8 h. Carcass stored at room temperature (18–22°C). Hematoxylin–eosin stain. **(A)** Frontal cortex, 100x magnification. **(B)** Cingulate cortex, 100x magnification. **(C)** Neuropil, 200x magnification. **(D)** Caudate-putamen, 100x magnification. **(E)** Corpus callosum, 100x magnification. **(F)** Hippocampus, 100x magnification. **(G)** Amygdala nuclei, 100x magnification. **(H)** Cerebellum, 100x magnification. **(I)** Choroid plexus, 200x magnification.

Eight hours postmortem, increased chromatin condensation and nuclear shrinkage in glial cells was observed throughout the brain (Figure 5; Supplementary Figure S5). Retraction spaces around blood vessels or within the Purkinje cell layer increased compared to previous time points. Granulation was observed in the neuropil of caudate-putamen, hypothalamus, and the white matter of the cerebellum from this necropsy time point onwards. Neurons of the cortex, thalamus, amygdala nuclei and reticular area nuclei started showing features of cytoplasmic dissolution while neurons of the septal nuclei and reticular area nuclei displayed mild nuclear fading. The cerebellum displayed moderate retraction spaces in the Purkinje cell layer and mild cytoplasmic dissolution and nuclear fading in Purkinje cells. Similarly, mild nuclear fading was observed in ependymal cells and choroid plexus epithelium. Mild axonal splitting and dissolution associated with myelin sheath dilation were recorded in the lateral trigeminal tract and cerebellum. The hippocampus showed less prominent axons in the CA1 and CA2 subfields and decreased staining in dark neurons. Moreover, decreased staining intensity in dark neurons was also observed in the cortex and hippocampus.

**Figure 6:**
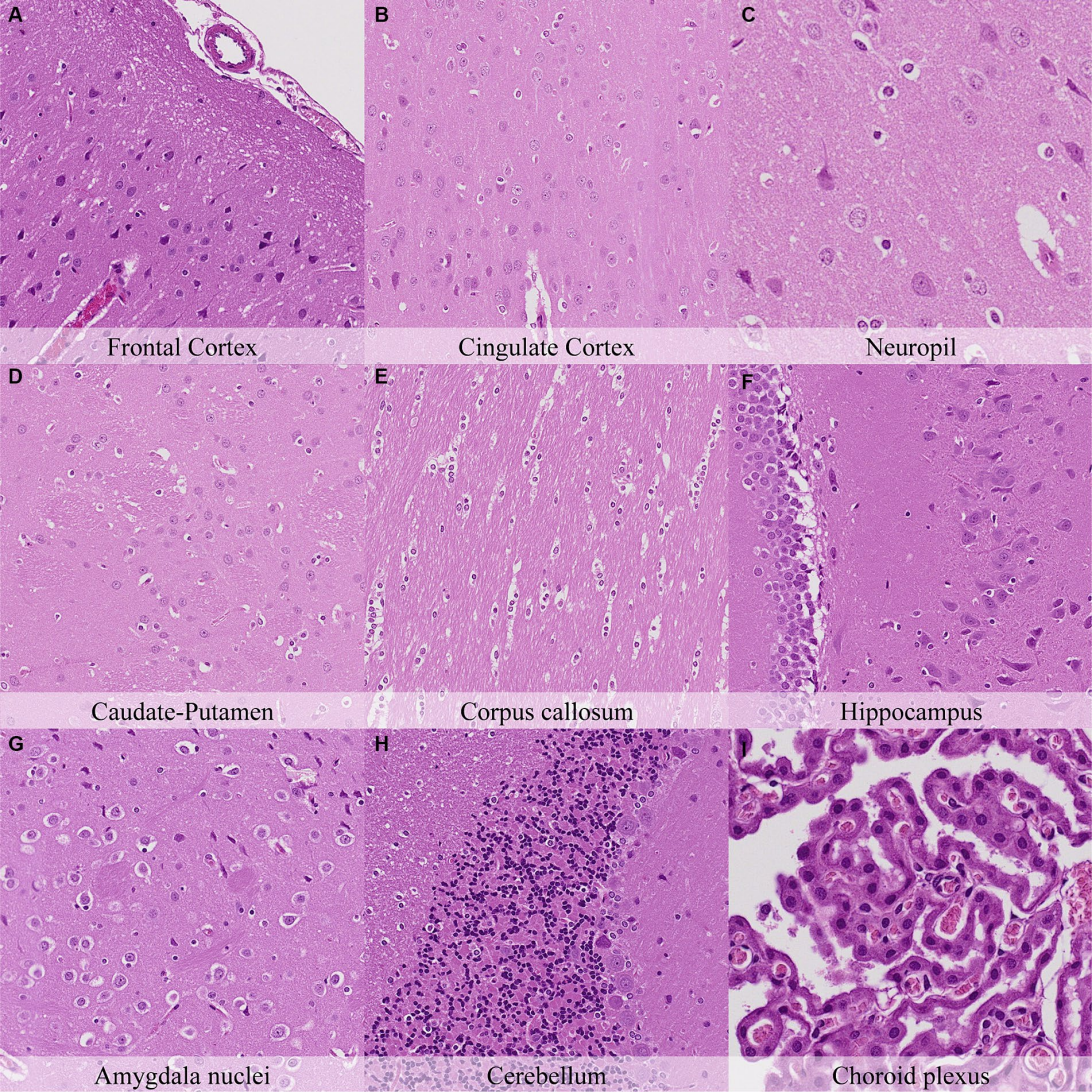
Postmortem microscopic changes in the brain after a delayed postmortem fixation of 12 h. Carcass stored at room temperature (18–22°C). Hematoxylin–eosin stain. **(A)** Frontal cortex, 100x magnification. **(B)** Cingulate cortex, 100x magnification. **(C)** Neuropil, 200x magnification. **(D)** Caudate-putamen, 100x magnification. **(E)** Corpus callosum, 100x magnification. **(F)** Hippocampus, 100x magnification. **(G)** Amygdala nuclei, 100x magnification. **(H)** Cerebellum, 100x magnification. **(I)** Choroid plexus, 200x magnification.

Twelve hours postmortem, glial cells showed differences in the intensity of the pericellular halo, which was moderate in the cortex, corpus callosum, caudate-putamen, septal nuclei, hypothalamus, and cerebellum and mild in the remaining neuroanatomical locations (Figure 6; Supplementary Figure S6). The cortex showed moderate granulation of the neuropil and an increased degree of cytoplasmic dissolution and nuclear fading in neurons. Similar findings were recorded in the neurons of remaining neuroanatomical locations. Moreover, axons in the cingulate cortex were slightly less prominent in contrast with earlier time points. Features of axonal splitting and dissolution associated with myelin sheath dilation increased at this time point and were moderate in the lateral trigeminal tract and mild in the predorsal bundle of the pons. Accordingly, marked trapezoid body microcavitation was recorded from this time point onwards.

**Figure 7:**
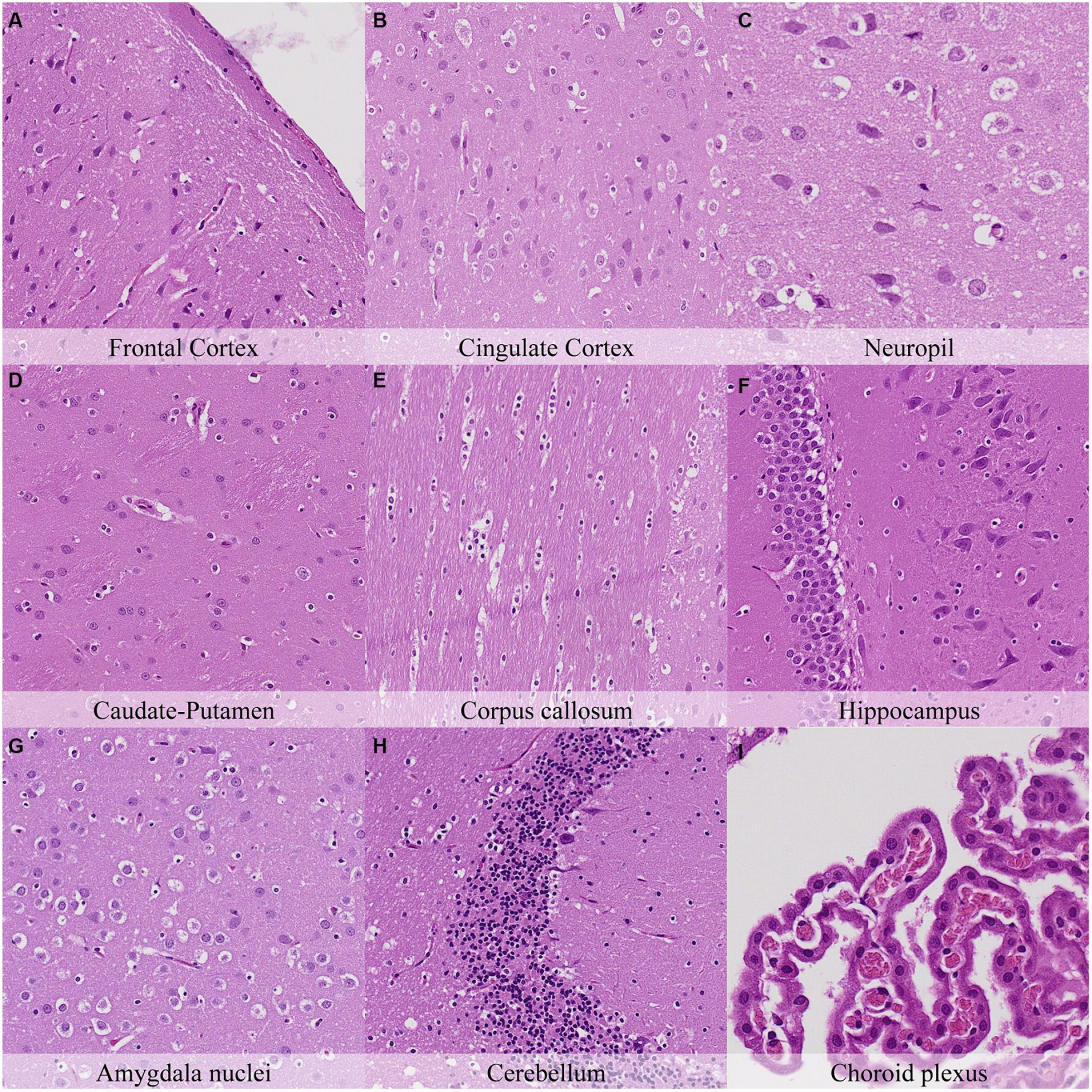
Postmortem microscopic changes in the brain after a delayed postmortem fixation of 24 h. Carcass stored at room temperature (18–22°C). Hematoxylin–eosin stain. **(A)** Frontal cortex, 100x magnification. **(B)** Cingulate cortex, 100x magnification. **(C)** Neuropil, 200x magnification. **(D)** Caudate-putamen, 100x magnification. **(E)** Corpus callosum, 100x magnification. **(F)** Hippocampus, 100x magnification. **(G)** Amygdala nuclei, 100x magnification. **(H)** Cerebellum, 100x magnification. **(I)** Choroid plexus, 200x magnification.

Twenty-four hours postmortem, nuclear shrinkage and chromatin condensation of glial cells were similar among all neuroanatomical locations examined and had marked intensity degree in most of them (Figure 7; Supplementary Figure S7). However, the pericellular halo in glial cells showed marked topographical differences as at the previous time point. Neuronal cytoplasmic dissolution was marked in the hypothalamus, amygdaloid nuclei, capsula interna and capsula externa from this time point onwards. In the cerebellum, the Purkinje cell layer displayed moderate cytoplasmic dissolution and nuclear fading while the granule cell layer showed marked nuclear shrinkage and mild pericellular halo. Moderate detachment from capillaries, cilial clumping, and cytoplasmic leaking were evinced in the choroid plexus. The leptomeninges showed minimal chromatin condensation, nuclear shrinkage, and detachment from neuropil.

**Figure 8:**
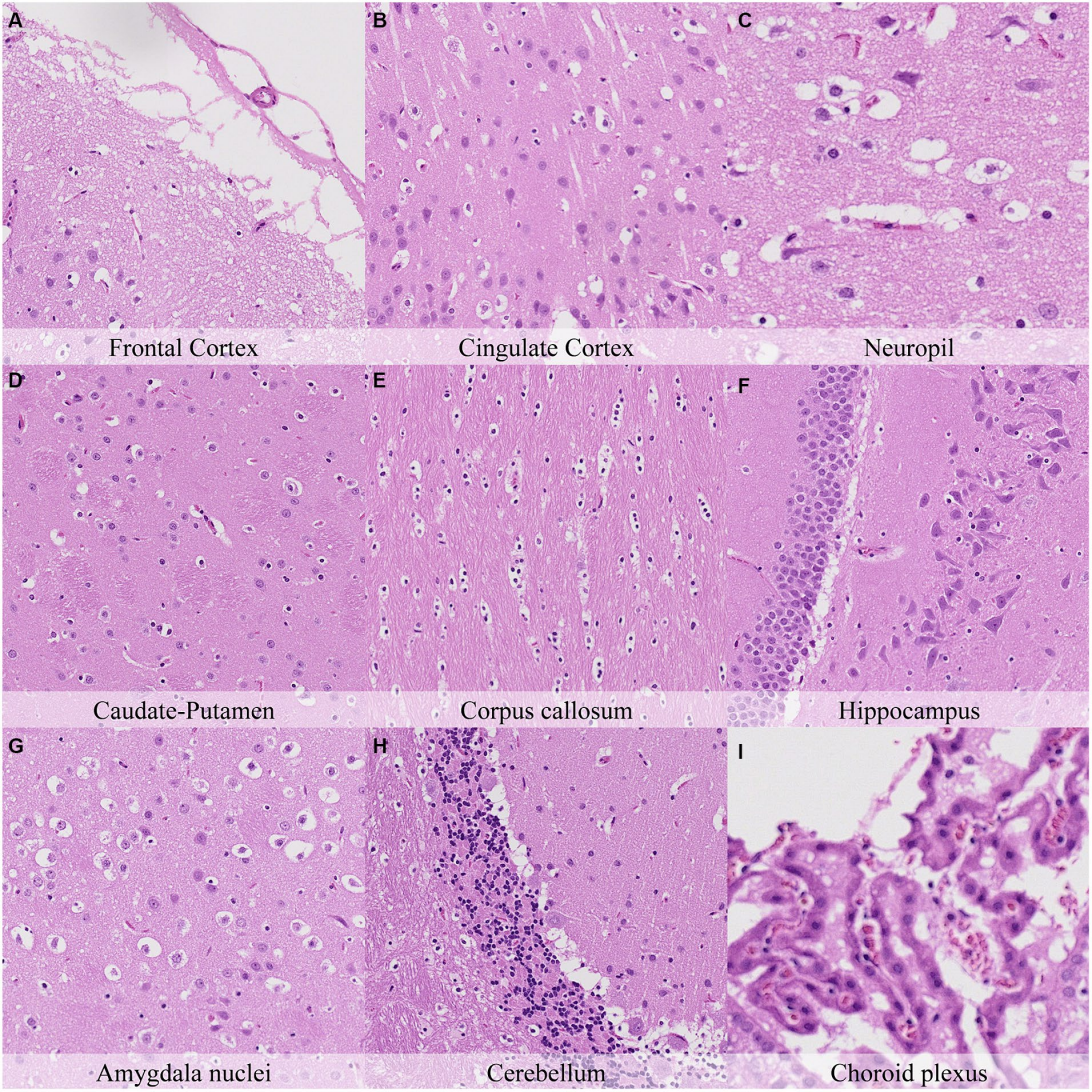
Postmortem microscopic changes in the brain after a delayed postmortem fixation of 36 h. Carcass stored at room temperature (18–22°C). Hematoxylin–eosin stain. **(A)** Frontal cortex, 100x magnification. **(B)** Cingulate cortex, 100x magnification. **(C)** Neuropil, 200x magnification. **(D)** Caudate-putamen, 100x magnification. **(E)** Corpus callosum, 100x magnification. **(F)** Hippocampus, 100x magnification. **(G)** Amygdala nuclei, 100x magnification. **(H)** Cerebellum, 100x magnification. **(I)** Choroid plexus, 200x magnification.

Thirty-six hours postmortem, at low magnification, a mild diffuse decrease in the staining affinity and fragmentation of the external surface were observed. At higher magnification, this fragmentation was associated with a diffuse increase in the granulation of the neuropil in the cerebral cortex that progressed towards microcavitation in the molecular layer (Figure 8; Supplementary Figure S8). In addition, the cortex showed a notable increase in the number of cells resembling dark neurons. This finding was more remarkable in regions that showed minimal or mild amounts of dark neurons at earlier time points (i.e., cingulate cortex and retrosplenial cortex). The hippocampus showed moderate retraction spaces in the blades of dentate gyrus together with decreased staining intensity of dark neurons and less prominent axons in CA1 and CA2 subfields compared to previous time points. Accordingly, increased retraction spaces were observed around blood vessels in the cerebral cortex and the Purkinje cell layer in the cerebellar folia. In the pons, moderate axonal splitting and dissolution and myelin sheath dilation were seen in the predorsal bundle. The choroid plexus showed marked cilial clumping and cytoplasmic leaking from this time point onwards.

**Figure 9:**
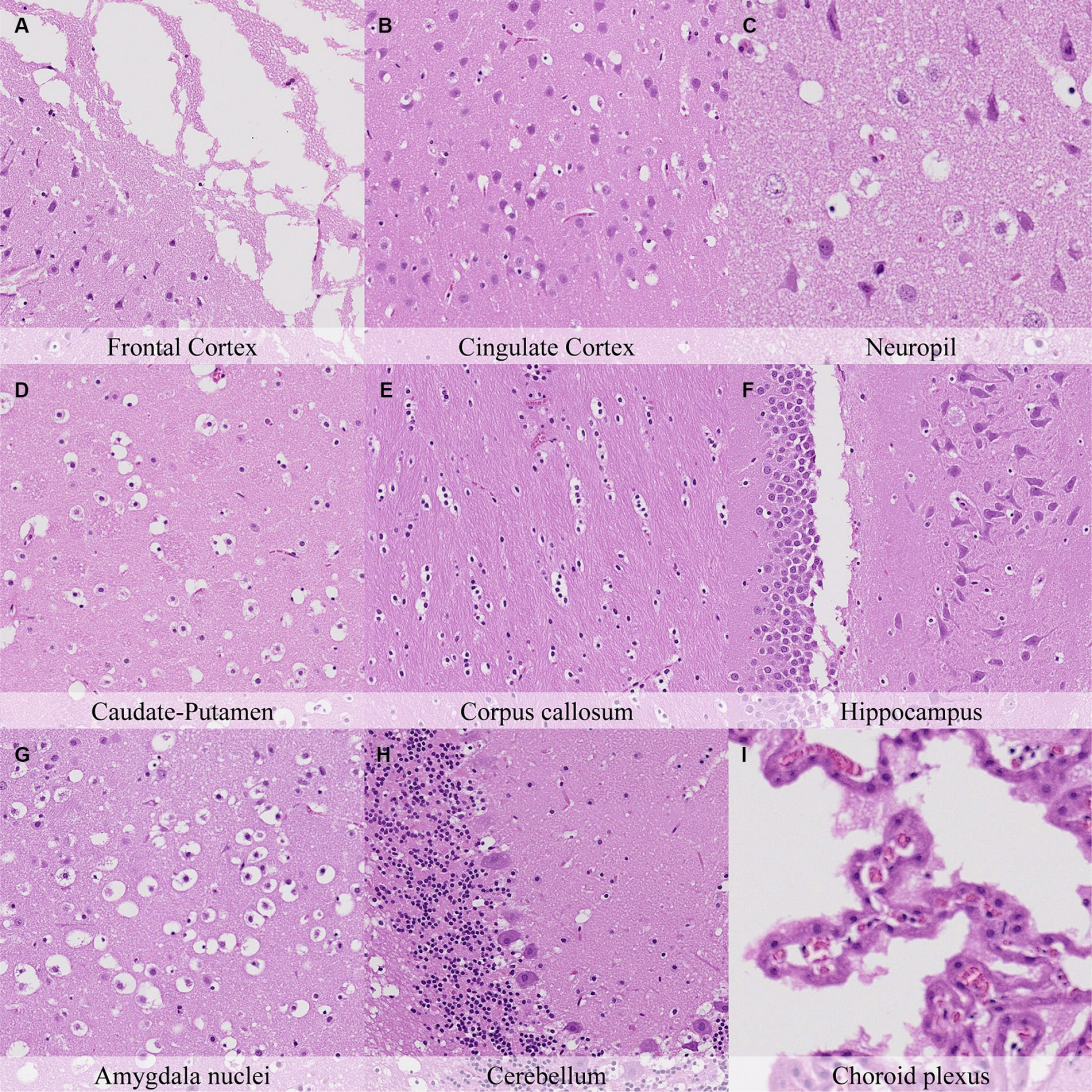
Postmortem microscopic changes in the brain after a delayed postmortem fixation of 48 h. Non-exsanguinated outbred RccHan^™^: WIST rat. Carcass stored at room temperature (18–22°C). Hematoxylin–eosin stain. **(A)** Frontal cortex, 100x magnification. **(B)** Cingulate cortex, 100x magnification. **(C)** Neuropil, 200x magnification. **(D)** Caudate-putamen, 100x magnification. **(E)** Corpus callosum, 100x magnification. **(F)** Hippocampus, 100x magnification. **(G)** Amygdala nuclei, 100x magnification. **(H)** Cerebellum, 100x magnification. **(I)** Choroid plexus, 200x magnification.

Forty-eight hours postmortem, fragmentation of the molecular layer of the cerebellar cortex increased with regards to the earlier time point and was also associated with marked granularity of the cortical neuropil (Figure 9). Accordingly, increased granularity of the neuropil or the white matter was recorded in the anterior commissure, capsula interna, capsula externa and cerebellum. The number of neurons closely resembling dark neurons in the cingulate and retrosplenial cortex was similar to that at 36 h postmortem. Glial cells in all neuroanatomical locations displayed marked nuclear shrinkage and chromatin condensation (Figure 9; Supplementary Figure S9). Pericellular halo intensity around glial cells was moderate in the anterior commissure, hippocampus, thalamus and internal capsula and external capsula, and marked at the remaining neuroanatomical locations.

### 3.3 Effect of exsanguination

**Figure 10:**
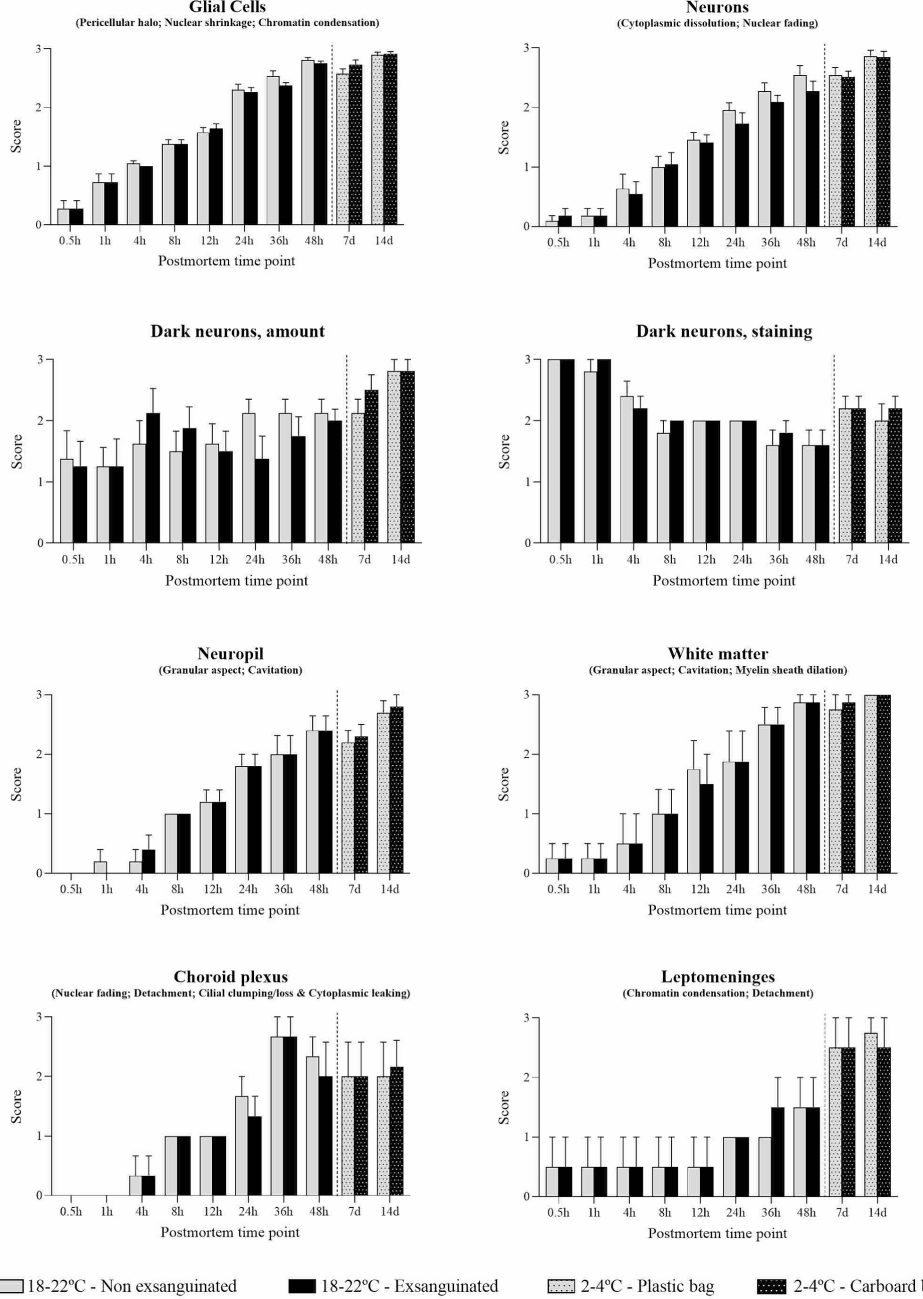
Summarized postmortem histological changes in the brain of exsanguinated and non-exsanguinated outbred RccHan^™^: WIST rats stored at room temperature (18–22°C) or refrigeration (2–4°C; stored in closed plastic bag or perforated carboard box) and necropsied at different time points after death.

The nature of histopathology changes in non-exsanguinated animals was identical to that in animals exsanguinated immediately after death. The complete histological evaluation of these animals is displayed in Supplementary Table S2. Occasional differences in the progression of these findings were observed without a clear trend (Figure 10). Postmortem changes in neurons (i.e., cytoplasmic dissolution and nuclear fading) showed slightly lower intensity in exsanguinated animals, mainly at later time points. This difference was observed in the brain cortex (8 to 48 h postmortem), caudate-putamen (12 h, 24 h and 48 h postmortem), hippocampus (24 h postmortem), thalamus (24 and 48 h postmortem), hypothalamus (36 h postmortem) and cerebellum (24 h postmortem). In addition, retraction spaces in the Purkinje cell layer were lower in exsanguinated animals 8 h postmortem. On the contrary, retraction spaces in the blades of the dentate gyrus were higher in exsanguinated animals 24 h postmortem (see Figure 10).

Glial cells showed chromatin condensation, nuclear shrinkage and pericellular haloes that increased in intensity over time. These findings occasionally showed slightly lower intensity in exsanguinated animals in some regions (i.e., brain cortex at 12 h, 36 h and 48 h; septal nuclei at 24 h; capsula interna and capsula externa at 36 h) but slightly higher in others (i.e., anterior commissure at 12 and 24 h; hippocampus at 12 h; reticular nuclear area at 12 h). Dark neurons were present at all time points with variations in their amount among neuroanatomical regions. As in non-exsanguinated animals, the number of cells morphologically resembling dark neurons was higher at later time points.

### 3.4 Effect of cooling, air circulation, body weight, and sex

**Figure 11:**
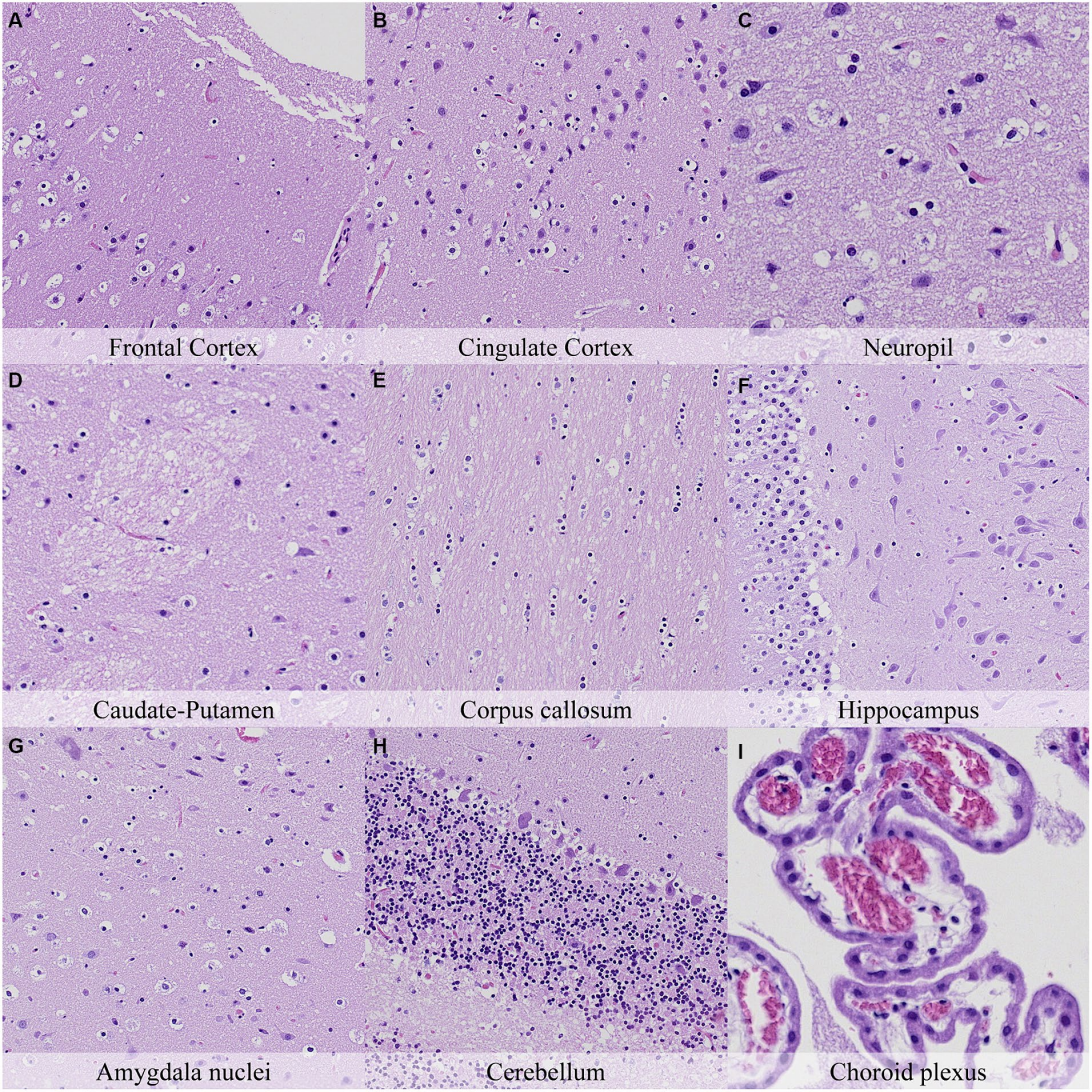
Postmortem microscopic changes in the brain after a delayed postmortem fixation of 14 days. Non-exsanguinated outbred RccHan^™^: WIST rat. Carcass stored under refrigeration (2–4°C). Hematoxylin–eosin stain. **(A)** Frontal cortex, 100x magnification. **(B)** Cingulate cortex, 100x magnification. **(C)** Neuropil, 200x magnification. **(D)** Caudate-putamen, 100x magnification. **(E)** Corpus callosum, 100x magnification. **(F)** Hippocampus, 100x magnification. **(G)** Amygdala nuclei, 100x magnification. **(H)** Cerebellum, 100x magnification. **(I)** Choroid plexus, 200x magnification.

**Figure 12:**
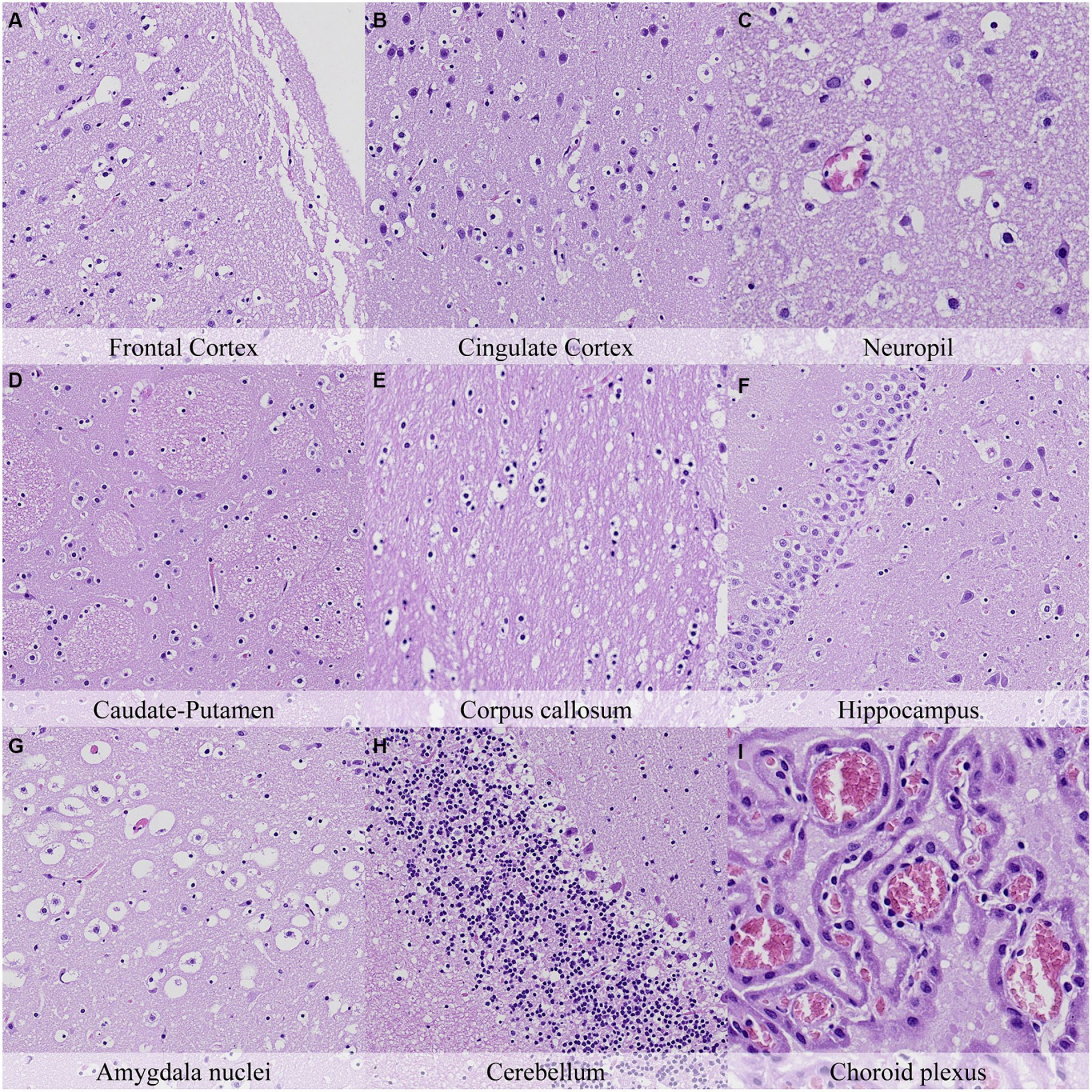
Postmortem microscopic changes in the brain after a delayed postmortem fixation of 7 days. Non-exsanguinated outbred RccHan^™^: WIST rat. Carcass stored under refrigeration (2–4°C). Hematoxylin–eosin stain. **(A)** Frontal cortex, 100x magnification. **(B)** Cingulate cortex, 100x magnification. **(C)** Neuropil, 200x magnification. **(D)** Caudate-putamen, 100x magnification. **(E)** Corpus callosum, 100x magnification. **(F)** Hippocampus, 100x magnification. **(G)** Amygdala nuclei, 100x magnification. **(H)** Cerebellum, 100x magnification. **(I)** Choroid plexus, 200x magnification.

Postmortem changes were recorded seven and 14 days after death (Figures 11, 12; Supplementary Table S3). The nature of the changes observed in animals stored under refrigeration was similar in animals stored at RT.

In general, the intensity of postmortem changes observed after seven days under refrigeration was slightly lower than those recorded after 48 h at room temperature (Figures 9, 11; Supplementary Figure S8, Supplementary Figure S9). For example, nuclear fading in neurons at several neuroanatomical locations showed similar intensity after seven days under refrigeration as after 48 h at RT. However, detachment from neuropil, chromatin condensation and nuclear shrinkage in leptomeninges were higher in animals refrigerated for seven days than in animals kept 48 h at room temperature. On the other hand, granulation and microcavitation of the neuropil in some regions (i.e., capsula interna, capsula externa and cerebellum) and retraction spaces in the Purkinje cell layer and blades of the dentate gyrus were similar in animals refrigerated for seven days and in animals kept at room temperature for 24 h. (Figures 7, 11; Supplementary Figure S6, Supplementary Figure S9).

The most advanced postmortem changes in the present study were recorded in animals after 14 days under refrigeration (Figure 12). At this time point, there was marked fragmentation of the external surface of the brain cortex associated with marked microcavitation of the molecular layer. Dark neuron amount increased at some neuroanatomical locations (i.e., cingulate and retrosplenial cortex) whereas basophilia of these dark neurons tended to decrease. Neurons showed marked cytoplasmic dissolution and nuclear fading in most examined areas except for the hippocampus and caudate-putamen. Similarly, the neuropil and white matter showed marked granulation and microcavitation in the cortex, hypothalamus, capsula interna, capsula externa and cerebellum and moderate granulation in the caudate-putamen. Glial cells showed marked pericellular haloes, nuclear shrinkage and chromatin condensation in the cortex, corpus callosum, septal nuclei, anterior commissure, hypothalamus, and reticular nuclear area and slightly less severe features in caudate-putamen, hippocampus, thalamus, capsula interna, capsula externa and cerebellum.

Although the weight of males was almost double that of females, few differences were found in the severity of postmortem findings that could be attributed either to sex or animal weight. Of note, some heavier animals showed slightly more severe postmortem changes in neurons and glial cells in some neuroanatomical compartments. No clear differences were observed among animals stored in a closed plastic bag and animals stored in a perforated carboard box to allow air circulation (Figure 10). Discrete differences were found in certain neuroanatomical locations without a clear trend.

## 4 Discussion

The CNS has a complex microanatomy and physiology. Moreover, the composition of the brain and its location within the cranial vault render it prone to artifacts, which can hinder histopathological evaluation (5). A well-known CNS artifact is the presence of autolysis, a postmortem change associated with delayed fixation (8). Forensic, diagnostic and toxicologic pathologists frequently face the challenge of differentiating autolytic changes from real findings. Moreover, autolytic changes can modify the performance of immunohistochemistry and histochemical stains (9–12). Despite its importance, investigation of microscopic postmortem changes is limited, with most of the studies covering only limited neuroanatomical locations, focusing on late stages of autolysis, or employing techniques other than light microscopy on routine H&E stains, such as transmission electron microscopy, immunohistochemistry, or biochemical analyses (11, 13–18). This work describes the onset and progression of autolytic changes in the brain of Wistar Han rats and provides rationale to properly identify them and optimize the histopathological evaluation.

The nomenclature proposed in the present work to characterize postmortem findings reflects a shared consensus among the 14 pathologists evaluating the slides. The terminology chosen aims to be simple and descriptive, applying whenever possible pre-existing terms already established in the pathology literature and avoiding the use of specific diagnostic terms (7, 8, 10, 16, 18, 19). Some terms employed in the present work (i.e., chromatin condensation, nuclear shrinkage, nuclear fading) reflect histological features that are also found in physiologic and pathologic processes of cell degeneration and/or cell death (e.g., pyknosis is evinced by chromatin condensation and shrinking; karyolysis is characterized by nuclear fading due to the action of endonucleases) (20–22). Indeed, after the somatic death of the organism, the cells remain alive for different periods of time. The lack of oxygen and nutrients and the accumulation of metabolic byproducts can result in cell injury leading to morphologic features similar to those observed in physiological/pathological processes of cell degeneration/death occurring *in vivo* (18). In this sense, nuclear shrinkage is a characteristic feature of apoptosis and caspase-independent cell death (20, 23). Moreover, nuclear size is influenced by mechanical forces, cytoskeletal integrity, and calcium homeostasis (24). These factors are also likely perturbed after somatic death of the organism (3, 25, 26).

The nature of histological changes was similar in all the animals of the study and increased in intensity over time, reinforcing the idea that postmortem changes are irreversible, predictable, and progressive (2). In forensic veterinary pathology, a major challenge during the systemic evaluation of postmortem changes is the inter-individual variability due to intrinsic (e.g., genetic background, age) and extrinsic (e.g., temperature, humidity) factors (27). In this regard, laboratory rodents represent an optimal model to evaluate these findings due to their genetic homogeneity and the tightly controlled environmental factors. Results obtained in the present work can likely be translated to other animal species or even humans (10, 16, 28–31). However, although the nature of postmortem changes may be shared among species, the onset and progression of these findings can differ due to intrinsic differences in body weight, cooling time, or metabolic activity of cells. For example, protein turnover and metabolic rate are 9.6 and 6.4 times higher, respectively, in rats than in humans, suggesting that autolytic changes could evolve more quickly in rodents (32). Besides the translatability of these results to other species, the present study provides a useful guide to properly estimate the postmortem interval in spontaneous deaths occurring in regulatory and investigational studies with rodents.

Postmortem changes differed among the major components and cell types of the CNS. Glial cells encompass a broad category of cells in the central nervous system with homeostatic functions and include astrocytes, oligodendrocytes, and microglia. In the present study, glial cells were evaluated all together due to their morphological similarities on routine H&E sections and the difficulties to accurately differentiate them at later time points after death (10). Glial cells showed pericellular haloes, chromatin condensation and nuclear shrinkage. Glial cells were similarly affected throughout the CNS and the sole postmortem finding that showed slight region-dependent differences was the pericellular halo. This finding was present at early time points in some regions and was more prominent in regions with higher densities of oligodendrocytes (i.e., corpus callosum, anterior commissure). Indeed, the retraction of the oligodendrocyte cytoplasm is a well-known artifact whereby oligodendrocytes acquire the so-called “fried-egg” or “honey-comb” appearance that helps distinguish oligodendrocyte-derived tumors (8).

Neurons showed two types of postmortem changes; most showed progressive cytoplasmic dissolution and nuclear fading whereas others acquired a dark-neuron-like appearance. Cytoplasmic dissolution and nuclear fading were minimal to absent at earlier time points and increased in intensity over time. These findings showed marked differences among neuroanatomical locations, likely associated with different neuronal metabolic activity at each location as well as different susceptibilities to oxidative stress and hypoxia (33, 34). In this sense, loss of cellular ATP secondary to oxygen deprivation is the main driver of oncosis (a non-regulated form of cell death) in cerebral ischemia due to alterations in the ionic balance and is considered the main pathway of postmortem cell death (18). Nuclear fading has been previously reported in the literature as “loss of nuclear basophilia” and is considered a reliable marker for autolysis and estimation of time after death (35–37). On the other hand, dark neurons were already present at earlier time points, and their amount was higher in some regions of the brain (i.e., frontoparietal cortex, hypothalamus, hippocampus) than in others (i.e., cingulate cortex). Dark neurons are a well-known CNS artifact characterized by intense amphophilic to basophilic staining, neuronal shrinkage, and irregular contours (38). They are associated with a variety of postmortem perturbations including traumatic handling of the brain, perfusion with cold fixative, short-term drying and immersion of the brain in saline, among others (39). Two main hypotheses of dark neuron formation point towards a depolarization of neurons and glutamate release leading to an alteration/contraction of cytoplasmic proteins that can be reversed after death by using proteases (40, 41). The increased amounts of dark neurons in animals necropsied at latter time points evinced the delayed fixation of the brain as another potential cause of their appearance. Moreover, the presence of dark neurons in animals with delayed postmortem fixation and their morphologic similarities with hypoglycemic neurons and/or ischemic neurons in peracute stages of degeneration may point towards the effect of reduced availability of oxygen and glucose and increased cellular waste products in neurons after somatic death (8, 42). Interestingly, in the present study the intensity of the staining of the dark neurons decreased slightly over time, which could support the hypothesis of an initial hypercontractibility of cytoplasmic proteins that is partially lost over time due to proteolysis of cytoskeletal proteins (8, 11, 18, 29, 43).

Retraction spaces around blood vessels, the blades of the dentate gyrus and the Purkinje cell layer were present at earlier time points and their severity increased slightly over time. These findings have been previously described in these locations as fixation artifacts and are potentially associated with swelling of astrocytic processes (8, 16, 44). The neuropil and white matter showed a granular aspect associated with microcavitation that increased in intensity over time. The white matter was more affected than the neuropil, especially the trapezoid body and the lateral trigeminal tract. This finding could be the result of widespread and fine postmortem vacuolation of the myelin sheaths (19, 45). Moreover, fragmentation of the external surface of the brain cortex was reported at later time points likely related to the progressive weakening of the neuropil, the physiological attachment of dura mater and periosteum and the mechanical forces exerted during necropsies (5).

Environmental conditions influence postmortem changes. In the present study, the onset and progression of these changes was notably slower in animals stored under refrigeration in contrast with animals stored at room temperature, corroborating the positive effect of refrigeration in delaying postmortem changes (46–48). Overall, severity of findings after 48 h at room temperature was higher than after seven days under refrigeration and similar to or slightly lower than after 14 days under refrigeration, confirming that higher environmental temperatures accelerate the progression of autolytic changes. Indeed, the parameters of time-after-death and temperature are responsible for 80% of body decomposition (49). Other parameters like humidity are also considered to play a minor role but this variable was not evaluated in the present study as no marked differences in the humidity were recorded.

Regarding host-dependent parameters, no clear differences were observed related to the sex and/or body weight of the animals or their exsanguination status. Blood cell degradation after death is associated with morphologic changes that end in cell rupture and release of their substrates and enzymes (50). Based on the authors’ experience, inefficient exsanguination of animals could favor the progression of postmortem changes in solid organs such as liver and kidneys. However, in the present study only minor differences were found in the CNS between exsanguinated and non-exsanguinated animals at similar time points. The impact of exsanguination on the progression of postmortem changes and the protective role of the blood–brain barrier in the CNS needs to be further investigated. Similarly, minor differences were found in animals refrigerated in a closed plastic bag in comparison to animals refrigerated in a perforated carboard box. Based on the authors’ experience in necropsies of large domestic animals, animal storage in plastic bags was expected to accelerate the progression of autolytic changes. A potential explanation for the lack of differences could be the reduced weight of rats in contrast with domestic animals and the fact that they were refrigerated immediately after death, both factors leading to a quick drop in body temperature. Another explanation could be that both storage methods were analyzed at late time points (seven and 14 days after euthanasia) and different results might have been observed at earlier time points. Finally, body weight of animals could play a role as male animals had notably higher body weights than females and showed slightly higher intensity degrees in the postmortem changes observed. The relevance of the size and weight of animals in the progression of postmortem findings has been previously highlighted in studies with larger animal species (36, 51).

## 5 Conclusion

The present work provides a comprehensive assessment of the onset and progression of postmortem histological changes in the CNS of rat tissues, which are recognizable, specific, and evolve over time. Postmortem histopathological changes differ among the major components and cell types of the CNS. Refrigeration of animals after death slows down the onset and progression of postmortem changes. Further studies are needed to elucidate the role of host-dependent factors such as sex and body weight.

## Data availability statement

The original contributions presented in the study are included in the article/Supplementary material, further inquiries can be directed to the corresponding authors.

## Ethics statement

All animals employed in the present study were surplus naïve animals from regulatory studies approved by the Ethical Committee of the Generalitat de Catalunya (Departament d’Acció Climática, Alimentació y Agenda Rural) and licensed under ref. 10832. Requirements of the Spanish Policy for Animal Protection (RED118/2021 and RED1386/2018) and the European Union Directive 2010/63 on the protection of experimental animals were always fulfilled.

## Author contributions

KW: Conceptualization, Data curation, Formal analysis, Methodology, Supervision, Writing – review & editing. AD: Data curation, Formal analysis, Writing – review & editing. KK: Data curation, Formal analysis, Writing – review & editing. RK: Data curation, Formal analysis, Writing – review & editing. FM: Data curation, Formal analysis, Writing – review & editing. KM: Data curation, Formal analysis, Writing – review & editing. YO: Data curation, Formal analysis, Writing – review & editing. PO: Data curation, Formal analysis, Writing – review & editing. LP: Data curation, Formal analysis, Writing – review & editing. TR: Data curation, Formal analysis, Writing – review & editing. OR: Data curation, Formal analysis, Writing – review & editing. RS: Data curation, Formal analysis, Writing – review & editing. NW: Data curation, Formal analysis, Writing – review & editing. RV: Data curation, Formal analysis, Writing – review & editing. RD: Conceptualization, Data curation, Formal analysis, Methodology, Writing – original draft, Writing – review & editing.
