# Supplementary material for: Onset and progression of postmortem histological changes in the central nervous system of RccHan™: WIST rats
Source: Front Vet Sci. 2024 May 21;11:1378609. doi: 10.3389/fvets.2024.1378609 (PMC11149423; doi:10.3389/fvets.2024.1378609)

**Supplementary Figure S9. Postmortem microscopic changes in the brain after a delayed postmortem fixation of 7 days.** Non-exsanguinated outbred RccHan$^{TM}$: WIST rat. Carcass stored under refrigeration (2-4ºC). Hematoxylin-Eosin stain. (A) Anterior commissure, 100x magnification. (B) Hippocampus, 20x magnification. (C) Thalamus, 100x magnification. (D) Hypothalamus, 100x magnification. (E) Capsula interna, 100x magnification. (F) Predorsal bundle, 100x magnification. (G) White matter, 200x magnification. (H) Dark neurons (cortex), 400x magnification. I) Dark neurons (Purkinje cells), 200x magnification.

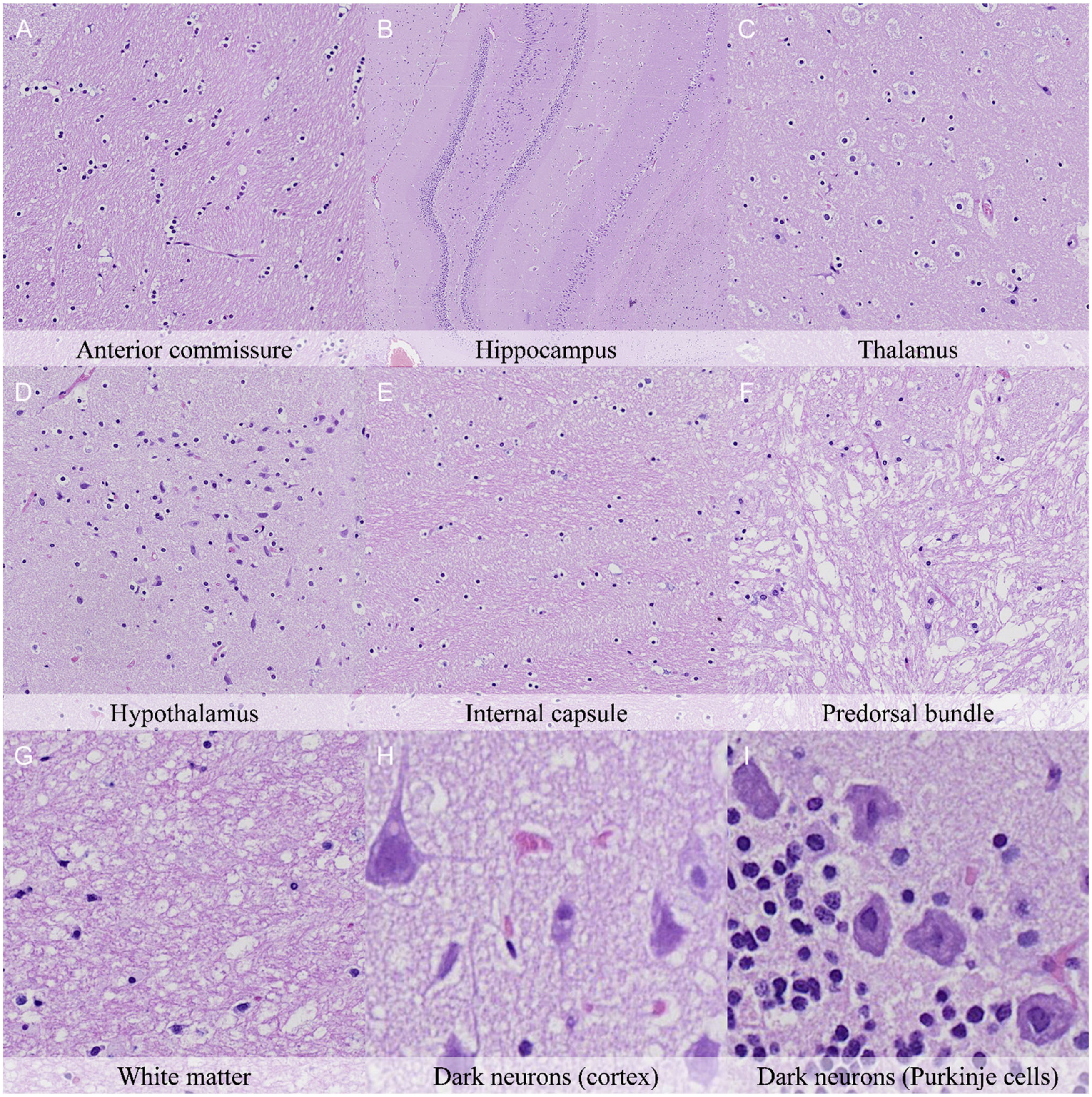

Supplement: Supplementary file 9 [file Image_9.pdf]
