# Supplementary material for: Onset and progression of postmortem histological changes in the central nervous system of RccHan™: WIST rats
Source: Front Vet Sci. 2024 May 21;11:1378609. doi: 10.3389/fvets.2024.1378609 (PMC11149423; doi:10.3389/fvets.2024.1378609)
Supplement: Supplementary file 11 [file Table_1.pdf]

 10.3389/fvets.2024.1378609

Supplementary Table S1: Time points and conditions evaluated during the study.

Temperature	Postmortem time	Sex	Exsanguination status	Storage
Room temperature (18-22 ºC)	0.5h	Female	Exsanguinated	Uncovered
			Non-exsanguinated	Uncovered
	1h	Female	Exsanguinated	Uncovered
			Non-exsanguinated	Uncovered
	4h	Female	Exsanguinated	Uncovered
			Non-exsanguinated	Uncovered
	8h	Female	Exsanguinated	Uncovered
			Non-exsanguinated	Uncovered
	12h	Female	Exsanguinated	Uncovered
			Non-exsanguinated	Uncovered
	24h	Female	Exsanguinated	Uncovered
			Non-exsanguinated	Uncovered
	36h	Female	Exsanguinated	Uncovered
			Non-exsanguinated	Uncovered
	48h	Female	Exsanguinated	Uncovered
			Non-exsanguinated	Uncovered
Refrigerator (2-4 ºC)	7d	Male	Non-exsanguinated	Plastic bag
				Carboard box
		Female	Non-exsanguinated	Plastic bag
				Carboard box
	14d	Male	Non-exsanguinated	Plastic bag
				Carboard box
		Female	Non-exsanguinated	Plastic bag
				Carboard box
