# Supplementary material for: Onset and progression of postmortem histological changes in the central nervous system of RccHan™: WIST rats
Source: Front Vet Sci. 2024 May 21;11:1378609. doi: 10.3389/fvets.2024.1378609 (PMC11149423; doi:10.3389/fvets.2024.1378609)
Supplement: Supplementary file 12 [file Table_2.pdf]

 10.3389/fvets.2024.1378609

Supplementary Table S2: Postmortem histological findings in the brain of exsanguinated and non-exsanguinated outbred RccHan^{TM}: WIST rats stored at room temperature (18-22 ºC) and necropsied at different time points after death. Post-mortem changes were scored from 0 to 3 (i.e., 0 = absent to minimal; 1 = mild; 2 = moderate; 3 = marked) based on their distribution, extension, and intensity degree.

	**NON-EXSANGUINATED**								**EXSANGUINATED**							
	**0.5h**	**1h**	**4h**	**8h**	**12h**	**24h**	**36h**	**48h**	**0.5h**	**1h**	**4h**	**8h**	**12h**	**24h**	**36h**	**48h**
**BRAIN CORTEX**																
Retraction spaces, around blood vessels	1	1	1	2	2	2	3	2	1	1	1	2	2	2	3	2
Neuropil, granulation/microcavitation	-	1	1	1	2	2	3	3	-	-	1	1	2	2	3	3
Dark neurons, amount, frontoparietal cortex	3	2	3	3	3	3	3	3	3	3	3	3	3	3	3	3
Dark neurons, amount, cingulate cortex	-	-	-	-	-	1	2	2	-	-	1	2	1	-	-	2
Dark neurons, amount, retrosplenial cortex	-	1	2	1	2	2	2	2	-	-	2	-	-	1	2	2
Dark neurons, amount, piriform cortex	2	2	2	2	2	3	3	3	2	1	3	2	2	-	2	2
Dark neurons, staining intensity	3	3	3	2	2	2	2	2	3	3	2	2	2	2	2	2
Prominent axons, cingulate cortex	3	3	2	2	1	1	1	1	3	3	2	2	1	2	1	1
Molecular layer, microcavitation	-	1	1	2	2	2	3 [a]	3 [a]	-	1	1	2	2	2	3 [a]	3 [a]
Glial cells, pericellular halo	1	1	1	1	2	3	3	3	1	1	1	1	1	3	3	3
Glial cells, nuclear shrinkage	-	-	1	1	2	2	3	3	-	-	1	1	1	2	2	2
Glial cells, chromatin condensation	-	-	1	2	2	3	3	3	-	-	1	2	2	3	3	3
Neurons, cytoplasmic dissolution	-	-	-	1	2	3	3	3	-	-	-	-	1	2	3	3
Neurons, nuclear fading	-	-	-	-	1	2	3	3	-	-	-	-	1	1	2	2
**CORPUS CALLOSUM**																
Glial cells, pericellular halo	1	1	1	2	3	3	3	3	1	1	1	2	3	3	3	3

Glial cells, nuclear shrinkage	-	-	1	1	1	2	2	3	-	-	1	1	1	2	2	3
Glial cells, chromatin condensation	-	-	1	3	2	3	3	3	-	-	1	3	2	3	3	3
**CAUDATE-PUTAMEN**																
Neuropil, granulation/microcavitation	-	-	-	1	1	2	2	2	-	-	-	1	1	2	2	2
Neurons, cytoplasmic dissolution	-	-	-	-	2	2	3	3	-	-	-	-	1	2	2	2
Neurons, nuclear fading	-	-	-	-	1	1	2	3	-	-	-	-	1	1	2	2
Glial cells, pericellular halo	-	1	1	1	2	2	2	3	-	1	1	1	2	2	2	2
Glial cells, nuclear shrinkage	-	-	-	1	1	1	2	3	-	-	-	1	1	1	2	3
Glial cells, chromatin condensation	-	-	1	2	2	3	3	3	-	-	1	2	2	3	3	3
**SEPTAL NUCLEI**																
Neurons, cytoplasmic dissolution	-	1	1	1	2	2	3	3	1	1	1	1	2	2	3	3
Neurons, nuclear fading	-	-	-	1	1	1	2	3	-	-	-	1	1	1	2	3
Glial cells, pericellular halo	-	1	1	1	2	3	3	3	-	1	1	1	2	2	2	3
Glial cells, nuclear shrinkage	-	-	1	1	2	2	3	3	-	-	1	1	1	1	2	3
Glial cells, chromatin condensation	-	-	-	1	1	3	3	3	-	-	-	1	1	3	3	3
**ANTERIOR COMMISSURE**																
Neuropil, granulation/microcavitation	-	-	-	1	1	1	1	2	-	-	-	1	1	1	1	2
Glial cells, pericellular halo	1	1	1	1	1	1	2	2	1	1	1	1	2	2	2	2
Glial cells, nuclear shrinkage	-	-	-	1	1	1	2	3	-	-	-	1	1	1	2	3
Glial cells, chromatin condensation	-	-	2	3	2	3	3	3	-	-	1	3	3	3	3	3
**HIPPOCAMPUS**																
Retraction spaces, blades of dentate gyrus	1	1	1	1	2	1	2	3	1	1	1	1	2	2	2	3

Retraction spaces, around blood vessels	1	1	1	2	2	2	2	2	1	1	1	2	2	2	2	2
Dark neurons, amount	2	2	2	2	2	2	2	2	2	2	3	2	2	2	2	2
Dark neurons, staining intensity	3	3	3	2	2	2	1	1	3	3	2	2	2	2	1	1
Prominent axon, CA1/CA2 regions	3	3	3	2	2	2	1	1	3	3	3	3	3	2	1	1
Neurons, cytoplasmic dissolution	-	-	1	1	1	2	2	2	-	-	1	1	1	1	2	2
Neurons, nuclear fading	-	-	-	-	1	1	1	1	-	-	-	-	1	1	1	1
Glial cells, pericellular halo	-	1	1	1	1	2	2	2	-	1	1	1	2	2	2	2
Glial cells, nuclear shrinkage	-	-	1	1	1	2	2	3	-	-	1	1	1	2	2	3
Glial cells, chromatin condensation	-	-	1	2	2	3	3	3	-	-	1	2	2	3	3	3
**THALAMUS**																
Dark neurons, amount	1	1	2	1	1	2	1	1	1	1	2	2	1	1	1	1
Dark neurons, staining intensity	3	3	2	2	2	2	2	1	3	3	2	2	2	2	2	1
Neurons, cytoplasmic dissolution	-	-	-	1	1	2	2	3	-	-	-	1	1	1	2	2
Neurons, nuclear fading	-	-	-	-	-	1	1	2	-	-	-	-	-	1	1	1
Glial cells, pericellular halo	-	1	1	1	1	2	2	2	-	1	1	1	1	2	2	2
Glial cells, nuclear shrinkage	-	-	-	1	1	2	2	3	-	-	-	1	1	2	2	3
Glial cells, chromatin condensation	-	-	1	2	2	3	3	3	-	-	1	2	2	3	3	3
**HYPOTHALAMUS**																
Neuropil, granulation/microcavitation	-	-	-	1	1	2	2	2	-	-	1	1	1	2	2	2
Dark neurons, amount	3	2	2	2	2	2	2	2	2	3	3	3	2	2	2	2
Dark neurons, staining intensity	3	3	2	2	2	2	2	2	3	3	2	2	2	2	2	2
Neurons, cytoplasmic dissolution	-	-	1	1	2	3	3	3	-	-	1	1	2	3	2	3

Neurons, nuclear fading	-	-	-	-	-	2	2	2	-	-	-	-	-	2	2	2
Glial cells, pericellular halo	-	1	1	1	2	2	2	3	-	1	1	1	2	2	2	3
Glial cells, nuclear shrinkage	-	-	-	1	1	2	2	2	-	-	-	1	1	2	2	2
Glial cells, chromatin condensation	-	-	1	2	2	3	3	3	-	-	1	2	2	3	3	3
**AMYGDALOID NUCLEI**																
Neurons, cytoplasmic dissolution	-	-	-	1	2	3	3	3	-	-	-	1	2	3	3	3
Neurons, nuclear fading	-	-	-	-	1	2	2	2	-	-	-	-	1	2	2	2
**CAPSULA INTERNA & CAPSULA EXTERNA**																
Neuropil, granulation/microcavitation	-	-	-	1	1	2	2	3	-	-	-	1	1	2	2	3
Neurons, cytoplasmic dissolution	-	-	2	2	3	3	3	3	-	-	2	2	3	3	2	3
Neurons, nuclear fading	-	-	-	1	1	2	2	3	-	-	-	-	1	2	2	3
Glial cells, pericellular halo	-	-	1	1	1	2	2	2	-	-	1	1	2	2	2	2
Glial cells, nuclear shrinkage	-	-	-	1	1	2	3	3	-	-	-	1	1	2	2	3
Glial cells, chromatin condensation	-	-	1	2	2	3	3	3	-	-	1	2	2	3	3	3
**CEREBELLUM**																
Retraction spaces, Purkinje cell layer	1	1	1	2	2	2	3	3	1	1	1	1	2	2	3	3
White matter, granulation & microcavitation	-	-	-	1	1	1	2	3	-	-	-	1	1	1	2	3
Neurons, Purkinje cells, dark staining (dark neurons)	3	2	2	1	2	2	1	2	3	3	3	2	2	2	2	2
Neurons, Purkinje cells, cytoplasmic dissolution & nuclear fading	-	-	-	1	1	2	2	2	-	-	-	1	1	2	2	2
Neurons, granule cells, chromatin condensation	1	1	2	2	2	3	3	3	1	1	1	2	2	2	3	3
Neurons, granule cells, pericellular halo	-	-	-	-	-	1	1	1	-	-	-	-	-	1	2	1
Glial cells, pericellular halo	-	-	1	1	2	2	3	3	-	-	1	1	2	2	2	3

Glial cells, nuclear shrinkage	-	-	-	1	1	1	2	2	-	-	-	1	1	1	2	2
Glial cells, chromatin condensation	-	-	1	2	2	3	3	3	-	-	1	2	2	3	3	3
**PREDORSAL BUNDLE**																
Axons splitting/dissolution	-	-	-	-	1	1	2	3	-	-	-	-	1	1	2	3
Myelin sheath dilation & microcavitation	-	-	-	-	1	1	2	2	-	-	-	-	1	1	2	2
**TRAPEZOID BODY**																
Microcavitation	1	1	2	2	3	3	3	3	1	1	2	2	3	3	3	3
**LATERAL TRIGEMINAL TRACT**																
Axons splitting/dissolution	-	-	-	1	2	3	3	3	-	-	-	1	1	3	3	3
Myelin sheath dilation & microcavitation	-	-	-	1	2	2	3	3	-	-	-	1	1	2	3	3
**RETICULAR NUCLEAR AREA**																
Neurons, chromatin condensation	-	-	-	2	2	2	3	3	-	-	-	2	2	2	3	3
Neurons, nuclear fading	-	-	-	1	1	2	2	3	-	-	-	1	1	2	2	3
Glial cells, pericellular halo	-	-	1	1	1	2	2	3	-	-	1	1	2	2	2	3
Glial cells, nuclear shrinkage	-	-	-	1	1	2	2	2	-	-	-	1	1	2	2	2
Glial cells, chromatin condensation	-	-	1	2	2	3	3	3	-	-	1	2	2	3	3	3
**EPENDYMA**																
Nuclear fading	-	-	-	1	1	1	2	2	-	-	-	1	1	1	2	2
Detachment, from neuropil	1	1	2	2	2	2	2 [b]	2 [b]	1	1	1	2	2	2	2 [b]	2 [b]
Cilial clumping/loss	-	-	-	1	1	2	3	3	-	-	-	1	2	2	3	3
**CHOROID PLEXUS**																
Nuclear fading	-	-	-	1	1	1	2	2	-	-	-	1	1	1	2	2

Detachment, from capillaries	-	-	-	1	1	2	3	2	-	-	-	1	1	1	3	1
Cilial clumping/loss & cytoplasmic leaking	-	-	1	1	1	2	3	3	-	-	1	1	1	2	3	3
**LEPTOMENINGES**																
Chromatin condensation & nuclear shrinkage	-	-	-	-	-	1	1	1	-	-	-	-	-	1	1	1
Detachment, from neuropil	1	1	1	1	1	1	1	2	1	1	1	1	1	1	2	2

[a] = Fragmentation
[b] = Rupture/discontinuity
