# Supplementary material for: Onset and progression of postmortem histological changes in the central nervous system of RccHan™: WIST rats
Source: Front Vet Sci. 2024 May 21;11:1378609. doi: 10.3389/fvets.2024.1378609 (PMC11149423; doi:10.3389/fvets.2024.1378609)
Supplement: Supplementary file 13 [file Table_3.pdf]

 10.3389/fvets.2024.1378609

Supplementary Table S3: Postmortem histological findings in the brain of exsanguinated and non-exsanguinated outbred RccHanTM: WIST rats stored under refrigeration (2-4 ºC) and necropsied at different time points after death. Post-mortem changes were scored from 0 to 3 (i.e., 0 = absent to minimal; 1 = mild; 2 = moderate; 3 = marked) based on their distribution, extension, and intensity degree.

	**PLASTIC BAG**				**CARDBOARD BOX**			
	**7 days**		**14 days**		**7 days**		**14 days**	
	**F**	**M**	**F**	**M**	**F**	**M**	**F**	**M**
**BRAIN CORTEX**								
Retraction spaces, around blood vessels	2	N/A	3	3	3	3	3	3
Neuropil, granulation/microcavitation	3	N/A	3	3	3	3	3	3
Dark neurons, amount, frontoparietal cortex	3	N/A	3	3	3	3	3	3
Dark neurons, amount, cingulate cortex	2	N/A	3	3	2	N/A	3	3
Dark neurons, amount, retrosplenial cortex	2	N/A	3	3	2	3	3	3
Dark neurons, amount, piriform cortex	2	N/A	3	3	3	3	3	3
Dark neurons, staining intensity	2	N/A	2	1	2	2	2	2
Prominent axons, cingulate cortex	-	N/A	-	-	-	N/A	-	-
Molecular layer, microcavitation	2 [a]	N/A	3 [a]	3 [a]	3 [a]	3 [a]	3 [a]	3 [a]
Glial cells, pericellular halo	3	N/A	3	3	3	3	3	3
Glial cells, nuclear shrinkage	3	N/A	3	3	3	3	3	3
Glial cells, chromatin condensation	3	N/A	3	3	3	3	3	3
Neurons, cytoplasmic dissolution	3	N/A	3	3	3	3	3	3
Neurons, nuclear fading	3	N/A	3	3	3	2	3	3
**CORPUS CALLOSUM**								

Glial cells, pericellular halo	3	N/A	3	3	3	3	3	3
Glial cells, nuclear shrinkage	3	N/A	3	3	3	3	3	3
Glial cells, chromatin condensation	3	N/A	3	3	3	3	3	3
**CAUDATE-PUTAMEN**								
Neuropil, granulation/microcavitation	2	N/A	2	2	2	2	2	2
Neurons, cytoplasmic dissolution	2	N/A	3	3	2	2	3	3
Neurons, nuclear fading	3	N/A	3	3	3	3	2	3
Glial cells, pericellular halo	2	N/A	2	3	2	2	2	2
Glial cells, nuclear shrinkage	2	N/A	3	3	2	3	3	3
Glial cells, chromatin condensation	3	N/A	3	3	3	3	3	3
**SEPTAL NUCLEI**								
Neurons, cytoplasmic dissolution	3	N/A	3	3	3	3	3	3
Neurons, nuclear fading	3	N/A	3	3	3	3	3	3
Glial cells, pericellular halo	2	N/A	3	3	2	2	3	3
Glial cells, nuclear shrinkage	3	N/A	3	3	3	3	3	3
Glial cells, chromatin condensation	3	N/A	3	3	3	3	3	3
**ANTERIOR COMMISSURE**								
Neuropil, granulation/microcavitation	2	N/A	2	3	2	2	3	3
Glial cells, pericellular halo	2	N/A	3	3	3	3	3	3
Glial cells, nuclear shrinkage	3	N/A	3	3	3	3	3	3
Glial cells, chromatin condensation	3	N/A	3	3	3	3	3	3
**HIPPOCAMPUS**								

Retraction spaces, blades of dentate gyrus	1	N/A	1	1	1	1	1	3
Retraction spaces, around blood vessels	2	N/A	2	2	2	2	2	2
Dark neurons, amount	2	N/A	3	3	3	3	3	3
Dark neurons, staining intensity	2	N/A	2	1	2	2	2	2
Prominent axon, CA1/CA2 regions	1	N/A	1	1	1	1	1	1
Neurons, cytoplasmic dissolution	3	N/A	3	3	3	3	3	3
Neurons, nuclear fading	1	N/A	2	2	1	1	2	2
Glial cells, pericellular halo	2	N/A	2	2	3	3	2	3
Glial cells, nuclear shrinkage	3	N/A	3	3	3	3	3	3
Glial cells, chromatin condensation	3	N/A	3	3	3	3	3	3
**THALAMUS**								
Dark neurons, amount	1	N/A	1	2	1	1	2	1
Dark neurons, staining intensity	2	N/A	2	2	2	2	2	2
Neurons, cytoplasmic dissolution	3	N/A	3	3	3	3	3	3
Neurons, nuclear fading	2	N/A	3	3	2	2	3	3
Glial cells, pericellular halo	2	N/A	2	2	3	3	2	3
Glial cells, nuclear shrinkage	2	N/A	3	3	3	2	3	3
Glial cells, chromatin condensation	3	N/A	3	3	3	3	3	3
**HYPOTHALAMUS**								
Neuropil, granulation/microcavitation	2	N/A	3	3	2	3	3	3
Dark neurons, amount	3	N/A	3	3	3	3	3	3
Dark neurons, staining intensity	2	N/A	2	2	2	2	2	2

10.3389/fvets.2024.1378609

Neurons, cytoplasmic dissolution	3	N/A	3	3	2	3	3	3
Neurons, nuclear fading	2	N/A	3	3	2	2	3	3
Glial cells, pericellular halo	2	N/A	3	3	3	3	3	3
Glial cells, nuclear shrinkage	2	N/A	3	3	2	2	3	3
Glial cells, chromatin condensation	3	N/A	3	3	3	3	3	3
**AMYGDALOID NUCLEI**								
Neurons, cytoplasmic dissolution	3	N/A	3	3	3	3	3	3
Neurons, nuclear fading	3	N/A	3	3	2	3	3	3
**CAPSULA INTERNA & CAPSULA EXTERNA**								
Neuropil, granulation/microcavitation	2	N/A	3	3	2	2	3	3
Neurons, cytoplasmic dissolution	3	N/A	3	3	3	3	3	3
Neurons, nuclear fading	2	N/A	3	3	2	2	3	3
Glial cells, pericellular halo	2	N/A	2	2	2	2	2	3
Glial cells, nuclear shrinkage	2	N/A	3	3	2	2	3	3
Glial cells, chromatin condensation	3	N/A	3	3	3	3	3	3
**CEREBELLUM**								
Retraction spaces, Purkinje cell layer	2	N/A	2	2	2	2	2	2
White matter, granulation & microcavitation	2	N/A	3	3	3	2	3	3
Neurons, Purkinje cells, dark staining (dark neurons)	3	N/A	3	3	3	3	3	3
Neurons, Purkinje cells, cytoplasmic dissolution & nuclear fading	2	N/A	3	3	3	2	3	3
Neurons, granule cells, chromatin condensation	3	N/A	3	3	3	3	3	3
Neurons, granule cells, pericellular halo	1	N/A	1	1	1	1	1	1

Glial cells, pericellular halo	2	N/A	3	3	2	2	3	3
Glial cells, nuclear shrinkage	2	N/A	3	3	2	2	3	3
Glial cells, chromatin condensation	3	N/A	3	3	3	3	3	3
**PREDORSAL BUNDLE**								
Axons splitting/dissolution	3	N/A	3	3	3	3	3	3
Myelin sheath dilation & microcavitation	3	N/A	3	3	3	3	3	3
**TRAPEZOID BODY**								
Microcavitation	3	N/A	3	3	3	3	3	3
**LATERAL TRIGEMINAL TRACT**								
Axons splitting/dissolution	3	N/A	3	3	3	3	3	3
Myelin sheath dilation & microcavitation	3	N/A	3	3	3	3	3	3
**RETICULAR NUCLEAR AREA**								
Neurons, chromatin condensation	3	N/A	3	3	3	3	3	3
Neurons, nuclear fading	3	N/A	3	3	3	3	3	3
Glial cells, pericellular halo	3	N/A	3	3	3	3	3	3
Glial cells, nuclear shrinkage	2	N/A	3	3	2	2	3	3
Glial cells, chromatin condensation	3	N/A	3	3	3	3	3	3
**EPENDYMA**								
Nuclear fading	1	N/A	1	2	1	1	1	3
Detachment, from neuropil	1	N/A	2 [b]	2 [b]	2 [b]	2 [b]	2 [b]	3 [b]
Cilial clumping/loss	3	N/A	3	3	3	3	3	3
**CHOROID PLEXUS**								

Nuclear fading	2	N/A	2	2	2	2	2	2
Detachment, from capillaries	1	N/A	1	1	1	1	1	2
Cilial clumping/loss & cytoplasmic leaking	3	N/A	3	3	3	3	3	3
**LEPTOMENINGES**								
Chromatin condensation & nuclear shrinkage	2	N/A	2	3	2	2	2	2
Detachment, from neuropil	3	N/A	3	3	3	3	3	3

[a] = Fragmentation
[b] = Rupture/discontinuity
N/A = Not analyzed / Tissue not present
